# Four decades of mapping and quantifying neuroreceptors at work *in vivo* by positron emission tomography

**DOI:** 10.3389/fnins.2022.943512

**Published:** 2022-09-08

**Authors:** Albert Gjedde, Dean F. Wong

**Affiliations:** ^1^Department of Neuroscience, University of Copenhagen, Copenhagen, Denmark; ^2^Department of Neurology and Neurosurgery, McGill University, Montreal, QC, Canada; ^3^Translational Neuropsychiatry Unit, Department of Clinical Medicine, Aarhus University, Aarhus, Denmark; ^4^Department of Radiology, Psychiatry, Neurology, and Neuroscience, Mallinckrodt Institute of Radiology, Washington University, St Louis, MO, United States

**Keywords:** neuroreceptor pet, kinetics, molecular neuroscience, neurotransmision, positron emission tomography

## Abstract

Decryption of brain images is the basis for the necessary translation of the findings from imaging to information required to meet the demands of clinical intervention. Tools of brain imaging, therefore, must satisfy the conditions dictated by the needs for interpretation in terms of diagnosis and prognosis. In addition, the applications must serve as fundamental research tools that enable the understanding of new therapeutic drugs, including compounds as diverse as antipsychotics, antidepressants, anxiolytics, and drugs serving the relief of symptoms from neurochemical disorders as unrelated as multiple sclerosis, stroke, and dementia. Here we review and explain the kinetics of methods that enable researchers to describe the brain’s work and functions. We focus on methods invented by neurokineticists and expanded upon by practitioners during decades of experimental work and on the methods that are particularly useful to predict possible future approaches to the treatment of neurochemical disorders. We provide an overall description of the basic elements of kinetics and the underlying quantification methods, as well as the mathematics of modeling the recorded brain dynamics embedded in the images we obtain *in vivo*. The complex presentation to follow is necessary to justify the contribution of modeling to the development of methods and to support the specifications dictated by the proposed use in clinical settings. The quantification and kinetic modeling processes are equally essential to image reconstruction and labeling of brain regions of structural or functional interest. The procedures presented here are essential tools of scientific approaches to all conventional and novel forms of brain imaging. The foundations of the kinetic and quantitative methods are keys to the satisfaction of clinicians that actively engage in treating the neurochemical disorders of mammalian brains in the fields of neurology, neurosurgery, and neuropsychiatry.

## Introduction

### Definition of compartments as tracer states

We designed the present text to consolidate the many studies and works of kinetic modeling that we reported since the early 1980’s. These works remain relevant to procedures commonly used in academic centers and commercial kinetic modeling software. While most of the descriptions focus on dynamic modeling by positron emission tomography (PET), we strive to generalize the description of procedures that best conform to models of multiple compartments. We hope that readers at all levels of knowledge of, and experience with, compartmental concepts will find the text to be a unified but critical compilation of standard compartment models involving regressions by linear, non-linear, or graphical methods. In the text, we provide the derivation of models of the relevant compartments, testable by methods used commonly and recently for neuroreceptor imaging and mapping by PET, consistent with the evolution of the last 40 years implied by the title of the text.

Some methods apply also to physiological processes active in heart, lung, and kidneys, and, therefore, are of more general utility. We attempt to address directly the very recently published comparisons of methods yielding evidence of occupancy of receptors and transporters with tracers without known regions of reference in the brain. We also discuss the still incompletely resolved issues of potential changes of plasma and brain protein binding that may yield paradoxical evidence of increased binding in the presence of expected inhibitors of radioligand binding to neuroreceptors. We hope to provide the reader with the experience in one common place not only of the historical origins of kinetic modeling but in particular of its current challenges and the opportunities for further progress of the quantification of neuroreceptor mapping.

Compartments have a definite mathematical definition, and a model is a family of compartments that simulate a biological system. The analysis of compartments tests the validity of models of kinetic interactions in single or multiple compartments and the results, by definition, predict quantitative interactions. The basic model is a hypothesis about the dynamics of a biological system of molecules that researchers test with predictions generated by the hypothesis. Nearly 75 years ago, Sheppard (1948) described compartments as quantities of a tracer or its metabolites that uniformly distribute in a compartmental space. We presume that each population of molecules adopts a single uniform state that changes over time but, by definition, is invariant in space, with a common reference to the number of molecules present in the space in molar units (i.e., per 6.02 × 10^23^). Rescigno and Beck (1972, 1987) modified Sheppard’s definition to relate it to a molecular state that changes over time in a manner regulated by the term,

(1) $$\frac{d\,m}{d\,t}=j-k\,m$$

where *m* is the quantity of tracer assigned to the compartment because of its state, *k* is the relaxation constant, and *j* refers to the flux of molecules into the compartment as a function of time. It follows from the definition that we obtain the relaxation constant from the relationship,

(2) $$k=\frac{j}{m}-\frac{1}{m}\,\left(\frac{d\,m}{d\,t}\right)$$

where, at steady-state, *dm/dt* = 0 and *k* is the turnover rate equal to *j/m.* According to the definition, the loss of tracer from the state (also known as the “relaxation” of the state) is a first-order process. Whether or not the requirement is fulfilled depends on the process responsible for the loss of the tracer from the compartment.

The more compartments it is necessary to assign to a model, the less the model distinguishes among the relevances of individual biological parameters. However, the model description of the observed data may be of practical value as a description of kinetic events. In addition, we can distinguish relaxation constants or transfer coefficients from many more or less superimposed spaces only when the constants are not of too different magnitudes. Slow expansion usually obscures rapid expansion as the events move toward equilibrium. Thus, by analyzing the organ uptake of a tracer as a function of time, we can identify only a limited number of compartments and transfer coefficients. In transient analysis, users obtain the magnitudes of the independent and dependent variables as functions of time and estimate the multiple coefficients from the relevant equations by linear or non-linear optimization of the model parameters from the data. The solution to the equations is a prototype of an operational equation commonly used for regression analysis. Using the equation above, we obtain the value of the parameter *k* by optimizing the equation relating the input (*j*) and tissue response (*m*) functions,

(3) $$m\,\left(T\right)=e^{-k\,T}\,\left[m\,\left(0\right)+\int_{0}^{T}j\,\left(t\right)\,e^{k\,t}\,d\,t\right]$$

where we aspire to assign an interpretation of biological interest to the parameter *k*, however, as a rule, the results of regression analyses do not relate easily to the biological characteristics of the system, and regression analyses commonly make sense only when the validity of a model is determined independently. Nonetheless, it usually is impossible to determine the validity of a model and, at the same time, obtain the most valid estimates of the parameters. Mathematical simulation of the model’s behavior and the actual experimental verification will help in the testing of the model.

### Determination of parameters from tracer states

Compartments are idealized descriptions of actual physiological processes and derive from a number of basic assumptions involving the even distribution of endogenous and exogenous molecules (the latter set up as tracers of native molecules such as glucose). The tracers rarely fulfill the assumptions completely, but reality often is close enough to yield useful results from the modeling. In general, when we quantify models of brain function using PET or single photon emission computed tomography (SPECT), the intravenously administered radioactive or labeled compounds move from the vascular source through the blood-brain barrier (BBB) to the compartments of the brain tissue. However, when we want to measure cerebral blood flow (CBF), we often (but not always) must assume that the brain is a single well-stirred space. When we measure oxygen consumption, we assume that the brain tissue is a single separate compartment in addition to the vascular volume. When the task is to measure glucose metabolism or binding potentials of neuroreceptors, we assume that there are multiple tissue compartments beyond the brain’s immediately accessible vascular volume. The tissue compartments include the sites where ligands occupy receptors responsible for populations of non-specifically and specifically bound molecules, respectively. Combinations of transport, enzyme, and receptor spaces of some tracers often lead to further complications of the models.

Regardless of the model of specific brain dynamics, the purpose of modeling is ultimately to connect the spaces and describe the connections in mathematical equations. The equations are used to extract the relevant parameters, such as clearance of tracer molecules from the circulation to brain tissue across the BBB (*K*_1_), the rate constant for the flux from brain tissue back to the vascular source (*k*_2_), the constant of binding to receptors in the brain (*k*_3_), and the rate of dissociation from the receptors (*k*_4_). Biological variables of physiological or pathophysiological significance, such as the absolute rate of CBF, link individually or in combination to the model parameters. For example, in the cases of blood-brain transfer coefficients, the magnitude of the unidirectional clearance parameter *K*_1_ is equal to the product of the fraction of the one-way extraction (*E*) of the radiotracer and the blood flow rate (*F*). In the cases of receptor binding, the ratio *k*_3_/*k*_4_ is the binding potential, defined as an index of receptors available for binding. The index is proportional to the ratio between the maximum number of receptors that can bind the tracer and the effective Michaelis-Menten relaxation constant, considering the presence of competitors, i.e., a ratio that we refer to as *B*_max_/*K’*_D_ where *K’*_*D*_ represents the affinity in the presence of competitors. The purpose of the analysis is, therefore, to estimate the model parameters that define and describe the underlying biological structures. The task is to combine the parameter estimates to obtain the ultimate biological variables of interest.

The challenge is to obtain independent estimates of parameters, some of which are rate constants and some of which are volumes derived from clearances. Researchers typically obtain dynamic images at specific times of the PET or SPECT images that capture radioactive molecules extracted from the capillary space of the brain tissue. Receptors, enzymes, or transporters, separately or together in combinations of two or three, interact with the tracer molecules, some of which return to the bloodstream. The researchers compare dynamic recording times vs. radioactivity measurements with predictions of specific compartment models as they solve the appropriate linear differential equations. The resulting parameter values optimize the fit between the predicted and measured time courses of labeled molecules. The ranges of biological variables, such as CBF, glucose and oxygen metabolism, and receptor availability, are the result of combinations of parameter estimates determined by mathematical analysis of biological processes.

## Compartmental modeling

### Single compartment modeling

By assigning a single compartment to the vascular and tissue elements, researchers obtain a record of the fate and distribution of the molecules of a labeled or detectable tracer that undergo immediate and even distribution in a single well-stirred pool of the tracer in the image pixel or region of interest forming the relevant regional or total brain volumes, depending on solubility. For the right tracers, only blood flow limits the uptake of the molecules by the brain, the distribution in the brain, and the subsequent removal of the molecules from the brain. Clinician researchers then derive the desired estimates of blood flow rates from the records of the fate of the tracer in the brain. This is possible because the single-compartment solution to the differential equations predicts an immediate and unlimited distribution in the combined vascular and brain tissue volumes occupied by the tracer (Gjedde, 2008),

(4) $$M^{*}\,\left(T\right)=F\,\int_{0}^{T}C_{a}^{*}\,\left(t\right)\,e^{-F\,\left(T-t\right)/V}\,d\,t$$

where M*(T) is the blood flow tracer quantity recorded in the brain or regions of the brain at a certain time *T* after administration. As defined in Table 1, among other common symbols, *F* is the blood flow rate, $C_{a}^{*}\,\left(t\right)$ is the arterial concentration of the tracer as a function of time, and *V* is the steady-state volume of distribution of the tracer in the relevant combined vascular and brain tissue volumes. The problem is that very few tracers match the ideal requirements of the single compartment modeling.

**TABLE 1:** Basic neurobiological variables and symbols.

Basic neurobiological variables	Symbol	Units
Cerebral blood flow	*F*	ml/100 g/min
Cerebral metabolic rate for oxygen	*CMR*_*O*2_	mol/100 g/min
Cerebral metabolic rate for glucose	*CMR*_*glc*_	mol/100 g/min
FDOPA conversion to fluorodopamine	*k*_3_	min^–1^
Net clearance	*K*_*in*_	ml/100 g/min
Receptor density	*B*_max_	pmol/g
Binding potential or receptor availability	*BP*_*ND*_	Ratio
Receptor half-saturation or Michaelis constant	*K*_D_	pmol/ml

#### Example 1: Determination of cerebral blood flow

Measurement of CBF rates and their functionally stimulated changes in the brain is a classic approach to functional brain mapping (Gjedde, 2008). In the first application of the approach, inhalation of a measurable tracer such as nitrous oxide, according to the methods of Kety and Schmidt (1948) and Raichle et al. (1983), allowed researchers to make measurements in humans. The methods replaced autoradiography of animal brains *ex vivo*, in which researchers stopped the circulation of the tracer after a single injection by sacrificing the animal and removing the brain. The corresponding event in studies of humans is the termination of sequential collections of tomographic images. The researchers validated the general approach in several studies that now represent standard methods of measuring blood flow to the brain. Although various applications are available, regional blood flow mapping requires arterial blood sampling for the mathematical extraction of the tissue response measured by PET or SPECT imaging (known as an “impulse”) to a brief input measured from arterial time-activity curves of rapid tissue entry and exit of the tracer. The input function is the key to the determination from measured data of the relevant parameters of the applicable compartment model that describes the uptake into a single brain space. Only a few tracers, such as labeled butanol or antipyrine, are freely diffusible to the extent required by the modeling (Gjedde et al., 1975; Gjedde, 1980). The more common tracer as radiolabeled water is less diffusible and thus requires researchers to include additional compartments in the modeling to account both for the uptake and for the radiolabeled water left behind in the circulation (Ohta et al., 1996).

### Dual compartment modeling

When two and only two compartments control the uptake and distribution of relevant tracer molecules in the brain, the molecules encounter only one significant obstacle or barrier to their entry into and occupation of the volume of distribution in brain tissue. The effect of this barrier is to both delay the distribution and expand the volume of distribution, depending on the properties of the barrier. The barrier can be any of several barriers to unlimited penetration, including membrane transporters, receptors, enzymes, or a combination of the above. Not only the blood flow but also the transport, binding, or metabolism of the tracer can delay the absorption, distribution, or subsequent removal of the labeled molecules from the brain volume occupied by the tracer. In the ideal cases, however, we can derive the kinetics of the tracer’s behavior by solution of the equation,


(5) $$M^{*}\,\left(T\right)=V_{0}\,C_{a}^{*}\,\left(T\right)+K_{1}\,\int_{0}^{T}C_{a}^{*}\,\left(t\right)\,e^{-K_{1}\,\left(T-t\right)/V_{e}}\,d\,t$$

where the relative size of the initial unidirectional clearance *K*_1_ and time *T-t* product and the tissue fluid distribution volume *V*_*e*_ determine whether any loss of tracer will be evident during the observation time. When the *K*_1_*(T-t)* product is very small compared to *V*_*e*_, or *V*_*e*_ very large compared to *K*_1_*(T-t)*, the equation reduces to the expression of irreversible trapping of the tracer in the brain tissue,


(6) $$M^{*}\,\left(T\right)=V_{0}\,C_{a}^{*}\,\left(T\right)+K_{1}\,\int_{0}^{T}C_{a}^{*}\,\left(t\right)\,d\,t$$

that reverts to the equation of the Gjedde-Patlak (GP), or Slope-Intercept (SI), plot (Gjedde, 1981),


(7) $$V\,\left(T\right)=\frac{M^{*}\,\left(T\right)}{C_{a}^{*}\,\left(T\right)}=V_{0}+K_{1}\,\int_{0}^{T}\frac{C_{a}^{*}\,\left(t\right)}{C_{a}^{*}\,\left(T\right)}\,d\,t=V_{0}+K_{1}\,\text{Θ}\,\left(T\right)$$

where the vascular space *V*_0_ is the ordinate intercept, *K*_1_ is the slope, and Θ(*T*) equals the normalized time integral of arterial tracer concentration (also see Equation 11 below), as defined by Gjedde (1981). Equation 7 is the original formulation of the Slope-Intercept Plot of blood-brain transfer of substances across the BBB that applies to uptake across a single barrier such as the BBB.

#### Example 2: Determination of cerebral oxygen consumption

Gaseous oxygen is subject to reduction by cytochrome α, α3 in the mitochondria of brain cells, where the oxygen undergoes rapid conversion to water. The conversion allowed early users of labeled oxygen with positron emission tomography (PET) to state that only two compartments of labeled molecules derive from oxygen gas in the brain, including labeled oxygen bound to circulating hemoglobin, and the resulting product in the form of labeled water in the tissue (Ter-Pogossian et al., 1970). Measurement of oxygen consumption (CMRO_2_) with oxygen-labeled oxygen gas was the first application of PET. The application rested on the predicted accumulation of labeled molecules, derived from the two-compartment solution to the two differential equations that describe oxygen’s reduction to water in the tissue and the subsequent accumulation and removal of labeled water, as described by Ohta et al. (1992). Thus, if *V*_0_ shown above is the volume of oxygen gas distribution in the cerebral arteries relative to the arterial concentration, then *K*_1_≡*CMRO*_2_/[*O*_2_] is the initial clearance (volume of removal per unit time) of oxygen from the vascular volume. The clearance is the result of significant labeled water production from the concentration of labeled oxygen gas in the arterial blood, and *k*_2_≡*K*_1_/*V*_*e*_ therefore becomes the rate of water removal from the exchange volume *V*_*e*_ = *K*_1_/*k*_2_.

### Triple compartment modeling

Three compartments (one vascular source and two tissue compartments) describe the uptake and distribution of the labeled or otherwise measurable tracer when several barriers delay the penetration into the entire brain volume, including, for example, two membrane transport processes or a membrane transport step plus a process of receptor binding or enzymatic reaction. The properties of the second tissue compartment in relation to the length of observation determine the extent to which we can detect this compartment kinetically. The properties also determine whether the third compartment effectively captures the labeled molecules. The accumulation of metabolites offers greater opportunities to quantify the uptake by means of different solutions to the equations (Gjedde et al., 2011), for example, when recording the brain uptake of tracers such as [^11^C]glucose, 6-[^18^F]fluorodeoxyglucose (FDG), or [^18^F]fluorodihydroxy-phenylalanine (FDOPA). The total radioactivity obeys a complex formula that incorporates the four constants defining three compartments,


$$M^{*}\,\left(T\right)=V_{0}\,C_{a}^{*}\,\left(T\right)$$

$$+K_{1}[\left(\frac{q_{2}-\left[k_{3}+k_{4}\right]}{q_{2}-q_{1}}\right)\int_{0}^{T}C_{a}^{*}\left(t\right)e^{-q_{2}\,\left(T-t\right)}dt$$

(8) $$+\left(\frac{q_{1}-\left[k_{3}+k_{4}\right]}{q_{2}-q_{1}}\right)\int_{0}^{T}C_{a}^{*}\left(t\right)e^{-q_{1}\,\left(T-t\right)}dt]$$

where $2\,q_{1}=k_{2}+k_{3}+k_{4}-\sqrt{{\left(k_{2}+k_{3}+k_{4}\right)}^{2}-4\,k_{2}\,k_{4}}$ and $2\,q_{2}=k_{2}+k_{3}+k_{4}+\sqrt{{\left(k_{2}+k_{3}+k_{4}\right)}^{2}-4\,k_{2}\,k_{4}}$, and where *k*_3_ and *k*_4_ are the rate constants for entering and leaving the second (or “internal”) tissue compartment as functions of the processes that determine entry and exit. The process can be any physical or biochemical step that delays further penetration of the labeled molecules, including passive or facilitated diffusion, active transport, binding, or enzymatic reaction. In some cases, the molecules penetrate but do not escape from the third compartment, which acts not only as a well but also as a trap, at least for some time.

In the cases when the rate constant *k*_4_ substantially is negligible with respect to the remaining rate constants, the total amount of labeled compound is modeled by means of the equation originally reported by Sokoloff et al. (1977),


$$M^{*}\,\left(T\right)=V_{0}\,C_{a}^{*}\,\left(T\right)$$

$$+K_{1}[\left(\frac{k_{2}}{k_{2}+k_{3}}\right)\int_{0}^{T}C_{a}^{*}\left(t\right)e^{-\left(k_{2}+k_{3}\right)\,\left(T-t\right)}dt$$

(9) $$+\left(\frac{k_{3}}{k_{2}+k_{3}}\right)\int_{0}^{T}C_{a}^{*}\left(t\right)dt]$$

This famous equation linearizes to an extension of the Slope-Intercept Plot that specifically addresses the trapping of the tracer in a second tissue compartment. The size of this second tissue compartment then depends on the magnitudes of *k*_2_ and *k*_3_, as presented by Gjedde (1982),


(10) $$M^{*}\,\left(T\right)≅\left(\frac{K_{1}\,k_{2}}{{\left[k_{2}+k_{3}\right]}^{2}}\right)\,C_{a}^{*}\,\left(T\right)+K_{1}\,\left(\frac{k_{3}}{k_{2}+k_{3}}\right)\,\int_{0}^{T}C_{a}^{*}\,\left(t\right)\,d\,t$$

where the ratio between the precursor in the first tissue compartment and the vascular source of the tracer eventually reaches an asymptotically fulfilled steady-state, and the volume of the tracer in the vascular bed becomes numerically negligible. The first part of the equation then describes a kinetically derived volume as the mathematically defined source of the tracer, *V*_*g*_ = *K*_1_*k*_2_/^(*k*_2_ + *k*_3_)2^. The second part of the equation describes the net clearance, *K* = *K*_1_*k*_3_/(*k*_2_ + *k*_3_), of the tracer into the trap of the second tissue compartment. Linear regression becomes possible when the magnitudes of the rate constants establish an apparent steady-state of the first tissue compartment (Gjedde, 1982, Patlak et al., 1983; Gjedde et al., 1985),


(11) $$V\,\left(T\right)=\frac{M^{*}\,\left(T\right)}{C_{a}^{*}\,\left(T\right)}≅V_{g}+K\,\int_{0}^{T}\frac{C_{a}^{*}\,\left(t\right)}{C_{a}^{*}\,\left(T\right)}\,d\,t=V_{\text{g}}+K\,\text{Θ}\,\left(T\right)$$

where *V*_*g*_ is the ordinate intercept, and *K* is the slope. It is evident that Equation (11) bears a superficial resemblance to Equation (7), but the parameters of the linear regression are different.

#### Example 3: Determination of cerebral glucose metabolism

We used radiolabeled native glucose derivatives as precursors of glucose phosphorylation to measure glucose metabolism in the brain. The first compartment is the distribution of a radiolabeled glucose analog in the vascular volume of the brain. Only a small fraction (∼10%) of the glucose analog in the cerebral circulation crosses the BBB and enters the brain tissue, where it occupies the first or precursor compartment in the tissue. The hexokinase enzyme then converts about one third of the glucose analog present in the tissue into the phosphorylated glucose-6-phosphate analog that remains trapped for some time in the second tissue compartment (Sokoloff et al., 1977; Reivich et al., 1979; Gjedde, 1982, Gjedde et al., 1985, 2011). When the task is to generate a quantitative map of regional glucose metabolism as a single image covering a time interval (often 45–90 min) after introduction of the tracer, the problem is that glucose transporters and glucose metabolic enzymes express different affinities for glucose, glucose analogs, precursors, and metabolites. Different affinities then affect the magnitude of Sokoloff’s tracer correction factor known as the Lumped Constant (LC). The LC itself really is not a constant but a variable derived from the ratio between the net extractions of a glucose analog and native glucose itself, measured and interpreted in several studies over several decades (Gjedde et al., 1985; Hasselbalch et al., 2001; Bass et al., 2011). Although the Lumped Constant varies significantly, depending on the effects of tissue homogeneity and altered blood glucose levels, it is possible to estimate the variable or altered values by full dynamic modeling, although the procedure rarely is used (Kuwabara et al., 1990).

#### Example 4: Determination of precursor metabolism and transmitter synthesis and loss in brain

Garnett et al. (1983) first visualized the conversion of DOPA to dopamine in the human brain by labeling DOPA with fluoro-18 as [^18^F]DOPA (FDOPA). The authors obtained the first PET images of trapped products in terms of residues of labeled fluorodopamine in the presynaptic vesicles of dopaminergic terminals, observed mainly in the striatum. Modeling the uptake of FDOPA posed particular problems because it depended on activities in multiple compartments. The problems arise from the conversion of FDOPA to 3-O-methyl-FDOPA at various sites in the body, including inside and outside the brain, as well as the outflow of a methylated product from the brain. However, the linearized Slope-Intercept Plot became popular as the simplest measure of the net tracer clearance *K* (see Equation 11), unfortunately often referred to as *K*_*i*_ in reports of the kinetics of FDOPA uptake, almost indistinguishable from *K*_1_ in texts, initially because of a misprint of the term *K*_*in*_. The estimates of *K*_*i*_ originally did not apply directly to any known flux because DOPA, unlike glucose in healthy volunteers, is the product of an enzymatic process in brain tissue rather than of a delivery from the circulation. This issue has led to searches for modeling techniques that provide estimates of the rate constant *k*_3_ of the trapping of fluorodopamine and subsequently for the efflux constant *k*_*loss*_ (Gjedde et al., 1991; Kumakura et al., 2005; Reith et al., 1994; Sigvard et al., 2022).

#### Example 5: Determination of receptor binding potential

Wagner et al. (1983) first visualized the binding of an exogenous radioligand to D_2_-like dopamine, and serotonin, receptors in the human brain, and Wagner’s group later reported that binding decreased with age (Wong et al., 1984, 1997a,1997b,1997c). The quantification of binding necessarily depends on the processes responsible for the binding of radioligands to the receptors that in turn reflects the affinity of the receptors for the ligand as an expression of the rate of dissociation of the ligand from the receptor. The first binding assays by PET involved the uptake in the human brain of the radioligand N-[^11^C]methylpiperone (NMSP), to which a receptor such as the dopamine D_2_ receptor has an affinity so high that binding remains substantially irreversible during the time available for tomography when tracers are labeled with the short-lived (20 min half-life) carbon-11 isotope. The binding is modeled according to Equations (10) and (11) rather than Equation (9) (Wong et al., 1986a,b), where regression analyses of the results by means of Equations (10) and (11) give estimates of the binding constant *k*_3_ as a function of the number of receptors available for binding.

**FIGURE 1:**
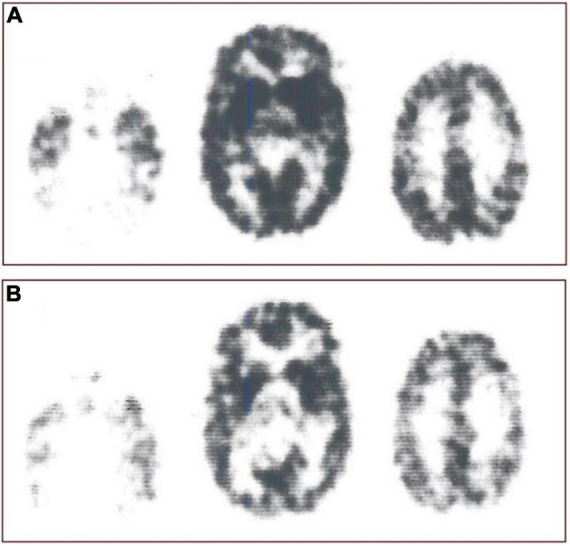
Receptor blockade. Study of interaction N-[^11^C]methylspiperone with exogenously administered unlabeled haloperidol as inhibitor of D_2_-like dopamine receptors from experiments reported by Wong et al. (1986b), showing original N-[^11^C]methylspiperone ([^11^C]NMSP) positron emission tomography sections before (**A**, top row) and after (**B**, bottom row) the ingestion of haloperidol. Images were obtained 45 min after injection of [^11^C]NMSP in both cases. From left to right in each row are the images from the three planes of the NeuroEcat camera (separated by 32 mm), demonstrating the temporal tips and cerebellar regions, basal ganglia, and cortical regions, and the frontoparietal region, scaled to the same intensity maximum and normalized for injected dose. Marked blockade of the uptake of the tracer in the basal ganglia is evident in center scan of Panel **(B)**.


Receptor blockade by endogenous or exogenous competitors, as shown in Figure 1 (adapted from Wong et al., 1986a), resulted in lower estimates of both net clearance *K* and binding rate constant *k*_3_ according to Equation (11) above rather than according to Equation (12) below. However, it is not possible to calculate binding potentials or absolute numbers of receptors without specific information on the value of the receptor dissociation rate symbolized by the term *k*_4_. The ratio of the rate constants *k*_3_ and *k*_4_ is the ratio of the amounts of bound and unbound ligands (B/F) in the Scatchard and Eadie-Hofstee plots that define the binding potential (*BP*_*ND*_) relative to non-displaceable (ND) tracer, also known as the receptor availability (Mintun et al., 1984). Researchers obtained estimates of the association and dissociation constants and their ratio (binding potential) only after the introduction of radioligands that undergo rapid but also reversible binding. The reversibility is due to the somewhat lower binding affinity than is the case for typical irreversibly binding ligands. The somewhat lower affinity is exemplified by the binding potentials determined for the dopamine D_2/3_ receptor ligand [^11^C]raclopride (Farde et al., 1986). We modeled the greater reversibility by means of Equation (8) that reverts to Equation (12) in the case of rapid binding when *V*_*e*_ then is replaced by *V*_*e*_ = *K*_1_/*k*_2_ (lately called *V*_*ND*_ for non-displaceable volume of distribution) at steady-state (Gjedde et al., 2005),

(12) $$V_{e}^{\prime }=K_{1}/k_{2}^{\prime }=\left[K_{1}/k_{2}\right]\,\left(1+\left(k_{3}/k_{4}\right)\right)=V_{e}\,\left(1+B\,P_{N\,D}\right)$$

where the volume of exchange depends on the steady-state distribution in regions of the brain with no specific binding. The earliest attempts to determine bound-free (B/F) ratios in animals *in vivo* by Kuhar et al. (1978) at a real or imagined steady-state inspired the later determinations of binding potentials simply from the ratios between tracer amounts in regions of interest and reference regions,


(13) $$B\,P_{N\,D}=\left[V_{e}^{\prime }/V_{e}\right]-1=\left[M_{R\,O\,I}^{*}/M_{N\,D}^{*}\right]-1$$

where ROI (regions-of-interest) commonly refers to sites of binding and the subscript ND as above refers to a site of non-displaceable tracer distribution in a volume with no specific (i.e., saturable) binding, also expressed by the B/F term.

#### Example 6: Determination of receptor binding capacity

**FIGURE 2:**
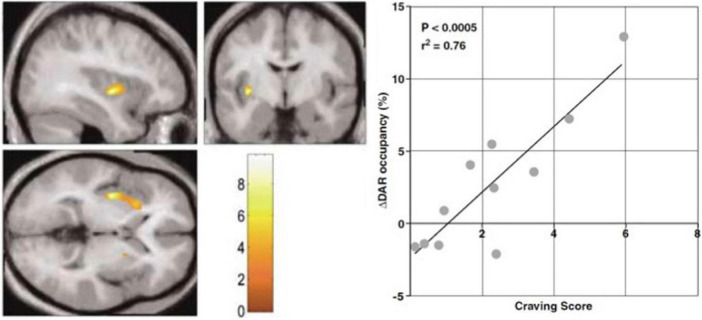
Endogenous release of intrasynaptic dopamine. PET study of [^11^C]raclopride binding during audiovisual cocaine craving cues. Abstinent cocaine abusing subjects received two consecutive PET imaging sessions, one with neutral cue during baseline PET, and one with the craving cue during second PET session. Statistical Parametric Mapping (SPM) showed clusters of volumes of interest with significantly increased dopamine release assumed to explain the changes of endogenous neurotransmitter occupancy of dopamine D_2_/D_3_ receptors in striatum. Left panel: SPM clusters in left anterior putamen where increased release of endogenous dopamine is alleged to raise dopamine receptor occupancy in response to craving cues with increased craving scores. Right panel: Scatterplot and regression of percent change of dopamine receptor (DAR) occupancy correlated with craving score for the cluster (adapted from Wong et al., 2006). Color scale shows correlation with craving score.

Expressed as a B/F ratio, the binding potential is a complex entity with a definition that is simple to understand but difficult to interpret. The assay depends on the concentration of at least two ligands, the endogenous ligand itself and the exogenous marker used in the experimental or clinical situation, and the local affinities of all relevant receptors for both ligands, exogenous as well as endogenous. Because there are many sites in brains at which concentrations of exogenous ligands can act, and affinities may differ, researchers have defined several binding potential terms that they can apply to different experimental situations. These terms relate to the compound’s concentration in the circulation relative to water (*BP*_*F*_) or plasma (*BP*_*P*_) or, as discussed above, to a brain tissue volume of no specific binding (*BP*_*ND*_) (Innis et al., 2007) that is closest to the original formulation and often the most relevant to the experimental or clinical application. It is also most relevant to the Scatchard or Eadie-Hofstee linearizations of the association of the amount of ligand bound at different concentrations and the binding potential,


(14) $$B=B_{\text{max}}-\left[V_{d}\,K_{D}\right]\,B\,P_{N\,D}$$

where *B* is the amount of bound ligand, *B*_max_ is the maximum binding capacity, and *V*_*d*_ is the volume of physical distribution of the radioligand. The volume *V*_*d*_ is also the volume to which the Michaelis half-saturation constant *K*_D_ applies, depending on the potential presence of additional inhibitors in the form of endogenous ligands such as dopamine or exogenous drugs such as the radioligand itself with a low specific activity. However, the exogenous ligand is not a real tracer if it has a concentration that measurably blocks the receptor and reduces the number of receptors unoccupied by ligands, as shown in Figure 2.

The binding must be in a steady-state for the rearrangement of the Michaelis-Menten Equation (Michaelis and Menten, 1913), shown linearized above as Equation (14), to apply. However, it is not necessarily the case that the estimates of *B*_max_ and *K*’_D_ will remain constant when exogenous ligand molecules are pharmacologically active and occupy the receptors. It remains the general assumption, however, when an unlabeled inhibitor competes with irreversibly binding PET radiotracers (Wong et al., 1986a,b,c, 1997a,b,c) at varying specific activities designed to yield estimates of *K*’_D_ and *B*_max_ as shown by Wong et al. (1998). To calculate values of *B*_max_ and *K*’_D_ (with unlabeled versions of the tracer) or the IC_50_ (concentration at 50% inhibition of an inhibitor that differs from the tracer), experiments require at least two PET sessions, without and with inhibition. This experimental design requires knowledge of the value of *V*_*e*_ (now often called *V*_*ND*_), sometimes extrapolated from multiple brain regions in the absence of a known or confirmed reference region in the brain (see Equations 60–65 below).

## Modeling brain receptor binding by arterial reference

### Tissue and arterial tracer quantities as functions of time

Neuroimaging of the behavior of tracers in the brain may need the simultaneous determination of tissue and arterial tracer quantities as functions of time. In the case of an irreversibly accumulating radioligand or —tracer, the ultimate objective of the analysis is to obtain an estimate of the rate of binding or metabolism (*k*_3_) of the tracer in structures of the brain. In the case of a reversibly accumulating radioligand, the ultimate object is to obtain the binding potentials (*BP*_*ND*_) and maximum binding capacities (*B*_max_) of brain receptors from total steady-state volumes of distribution of the tracer (V(T) or *V*_*T*_). To achieve this goal, one possibility is to determine the values of primary variables that form the basis of the GP-SI Plot. Originally, we used the GP-SI Plot to estimate the kinetic parameters of tissue tracer accumulation from irreversibly metabolized tracer substrates of brain hexokinase to obtain estimates of brain glucose consumption (*CMR*_*glc*_). When we plotted the findings in units of time vs. tracer uptake in arterial blood and brain tissue, we obtained estimates of the rate of metabolism from the slope, as well as estimates of the distribution volume from the intercept in a computationally convenient fashion (Gjedde, 1981, 1982; Patlak et al., 1983).

**FIGURE 3:**
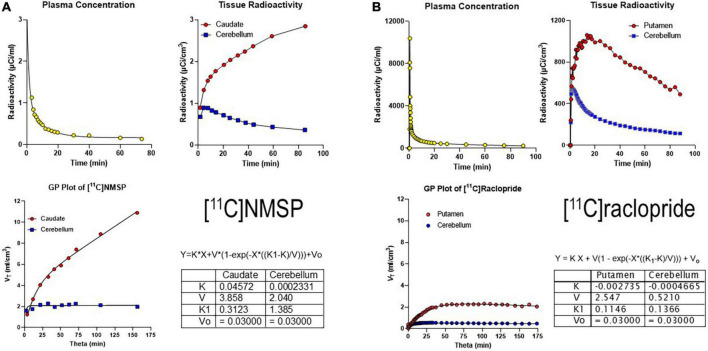
Irreversibly and reversibly binding radiotracers. **(A)** Gjedde-Patlak (GP) plot applied to the irreversibly binding radioligand [^11^C]NMSP. Upper left panel: Arterial plasma time-activity curve (TAC) corrected for metabolites. Upper right panel: Caudate and cerebellum TAC. Lower left panel: GP plot applied to total volume of distribution (*V*_*T*_) vs. normalized time (“see Equation 15”). Lower right panel: Parameter output by GP plot. **(B)** GP plot applied to reversibly binding radioligand [^11^C]raclopride. Upper left panel: Arterial plasma TAC corrected for metabolites. Upper right panel: Putamen and cerebellum TACs. Lower left panel: GP plot applied to *V*_*T*_ vs. Theta. Lower right panel: Parameter output by GP plot. Adapted from Gjedde (2003).

The GP-PI Plot records a dependent variable with a unit of volume of distribution vs. an independent variable with a unit of time. The dependent variable with a unit of volume is obtained from the tracer quantity in a brain region or structure relative to the radioactivity in arterial blood (yielding the volume of distribution commonly symbolized by *V*(T) or V_*T*_ as a function of time). The independent variable with a unit of time is obtained from the integral of the radiotracer concentration in the arterial circulation as a function of time relative to the tracer concentration in the arterial circulation at the specific time of determination (symbolized by Θ(T) with a unit of time). The two measures obey the definitions,


(15) $$V\,\left(T\right)\equiv \frac{m\,\left(T\right)}{c_{a}\,\left(T\right)}\;\Theta \,\left(T\right)\equiv \int_{0}^{T}\frac{c_{a}\,\left(t\right)\,d\,t}{c_{a}\,\left(T\right)}$$

where the two variables *m(t)* (tracer quantity in brain tissue) and *c*_*a*_*(t)* (tracer concentration in circulation) are measured simultaneously and continuously by imaging the brain and brain regions and by sampling arterial blood. The relation of the variable *V*(T) to the variable Θ(T) as dependent and independent variables, respectively, obeys an equation derived from Equation (9) as a function of the time variable Θ(T),


(16) $$V\,\left(T\right)=K\,\text{Θ}\,\left(T\right)+V_{\text{f}}\,\left(1-\exp\left(-\Theta \,\left(T\right)\,\left(\frac{K_{1}-K}{V_{\text{f}}}\right)\right)\right)+V_{0}$$

where the symbols have their conventional meaning. Regression then yields estimates of the parameters *K* (which is the net clearance of the radiotracer in the blood into the brain), *V*_*f*_ (precursor pool volume), *K*_1_ (unidirectional clearance from blood to the brain), and *V*_0_ (volume of blood in brain tissue). Figures 3A,B show examples of the results.

### Irreversible receptor binding of [^11^C]NMSP

The original PET imaging of dopamine D_2_/D_3_ receptors with N-[^11^C]methylspiperone ([^11^C]NMSP) in the living human brain happened in 1983 (Wagner et al., 1983). Subsequent investigations revealed that the numbers of dopamine D_2_/D_3_ receptors in the striatum fall with age and gender. However, the method comparing the specific-to- the non-specific binding ratio of the radioligand used by the investigators (Kuhar et al., 1978) was not sufficiently sensitive to reveal quantitative changes during the course of human health and disease.

To address this shortcoming, we successfully applied the GP-SI plot presented above to measure *CMR*_*glc*_ by analysis of PET images obtained with [^14^C]FDG, [^18^F]FDG, and 3-O-[^11^C]methylglucose (Gjedde, 1981, 1982), and to measure DOPA decarboxylase activity in PET images obtained with 6-[^18^F]fluoro-L-DOPA (Reith et al., 1994).

Figure 3A shows the results of the application of the SI plot to [^11^C]NMSP that revealed the irreversibility of the binding of NMSP to dopamine receptors. The application included measures of the radioactivity in arterial blood after arterial administration and the measured uptake values in the caudate nucleus and cerebellum (Wong et al., 1986a,1997a).

We also determined the resulting GP-SI plot of volumes of distribution as functions of the extended time variable Θ(*T*) (see Equation 9 above) from tracer concentrations in the arterial circulation (Figure 3A). The GP-SI plot also shows the equation of non-linear regression used to derive the outcome measures listed in the table of Figure 3A, showing the reversible binding to serotonin receptors and the irreversible binding to dopamine receptors.

### Reversible receptor binding of [^11^C]raclopride

The binding potential *BP*_*ND*_ is the quantity of bound tracer divided by the unbound tracer in the brain at equilibrium when the content of bound tracer quantity B is constant, i.e., when d*B*/dt = 0. The binding is not at equilibrium when the bound quantity *B* in the tissue varies, as it does during increases and decreases of binding due to delivery or washout or both, despite the standard correction for decline of radioactivity necessitated by the half-life of the tracer. It is a significant limitation of common reference region methods of analysis such as SRTM, Logan Plot, and MRTM_2_ (Lammertsma and Hume, 1996; Logan et al., 1996; Ichise et al., 2003) that do not take the absence of equilibrium into account as required by accurate determination of binding potentials. All current reference tissue methods of analysis fail to yield a precise measure of the quantity of unbound tracer at the time(s) of binding equilibrium. For all bolus injections of the radiotracer, including the bolus plus infusion administration (Carson et al., 1993), we typically find only one instance of equilibrium of the bound radioligand and most commonly at the peak of the binding. Infusion of the tracer fails to remedy the problem of absent equilibrium because the infusion typically is designed to achieve an unchanging value of the total quantity of the radiotracer in the brain tissue rather than a constant quantity of the bound tracer only. Most of the methods we commonly use to estimate the maximum quantity (*B*_max_) and the affinity (*K*_D_) of binding by reversible radioligands with a known reference region may suffer from this uncertainty.

A reversibly binding radioligand is expected to eventually reach a steady-state volume of distribution, unlike the irreversibly binding tracer [^11^C]NMSP. When applied to the reversibly binding radioligand [^11^C]raclopride, the result of the GP-SI plot is shown in Figure 3B.

Together, Panels A and B of Figure 3 above show irreversibly and reversibly binding radioligand quantities in brain tissue following arterial administration and circulation. While a reference region such as the cerebellum is shown for both tracers, the results of the GP-SI plot applications are independent of the presence or absence of the reference region.

**FIGURE 4:**
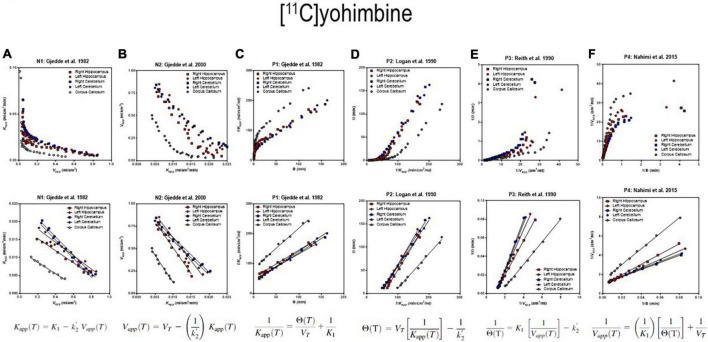
Receptor binding relative to arterial input functions. **(A**, Top) (Graph N1) inversely related plot of *y* vs. *x* for entire duration of PET. **(A**, Bottom) (Graph N1) negative slope plot of linear relation. **(B**, Top) (Graph N2) inversely related plot of *y* vs. *x* for entire duration of PET. **(B**, Bottom) (Graph N2) negative slope plot of linear relation. **(C**, Top) (graph P1) positively related plot of *y* vs. *x* for entire duration of PET. **(C**, Bottom) (graph P1) positive slope plot of linear relation. **(D**, Top) (Graph P2) positively related plot of *y* vs. *x* for entire duration of PET. **(D**, Bottom) (Graph P2) positive slope plot of linear relation. **(E**, Top) (Graph P3) positively related plot of *y* vs. *x* for entire duration of PET. **(E**, Bottom) (graph P3) positive slope plot of linear relation. **(F**, Top) (Graph P4) positively related plot of *y* vs. *x* for entire duration of PET. **(F**, Bottom) (Graph P4) positive slope plot of linear relation. Adapted from Nahimi et al. (2015).


The six separate sets of linear regressions in Figure 4 represent the results of the application of the different alternatives of linear regression applied to the data. We obtained quantitative results of reversible receptor binding by linear regression of the main variables of binding. We used six different linear regressions published separately by different authors. The six plots reveal variations of an approach to a linear relationship when the reversible binding reaches a secular steady-state of bound and unbound quantities of the radiotracer in brain tissue. We identified the six panels of Figure 4 by the symbols N1 and N2 for the two linear relationships with negative slope and P1-P4 for the four linear relationships with positive slope of the reversibly binding tracer [^11^C]yohimbine, as shown in Figures 4A–F. The six plots yielded the three main parameters of binding in combinations of two for each plot. We included the operative equation of each of the six linear plots in each of the six panels. We then used the six linear equations derived from the primary differential equations of the brain uptake of the radioligand (Nahimi et al., 2015).

(17) $$\frac{d\,m_{1}}{d\,t}=V_{o}\,\frac{d\,c_{a}}{d\,t}$$

(18) $$\frac{d\,m_{2}}{d\,t}=K_{1}\,c_{a}-k_{2}^{\text{ROI}}\,m_{2}$$

where *k*_2_ of the region of interest (ROI), $k_{2}^{\text{ROI}}$ (*k*_2_’ in Figure 4), is defined as,


(19) $$k_{2}^{\text{ROI}}=\frac{k_{2}}{1+B\,P_{\text{ND}}}=\frac{K_{1}}{V_{\text{ND}}\,\left(1+B\,P_{\text{ND}}\right)}=\frac{K_{1}}{V_{T}}$$

and *BP*_*ND*_ is the binding potential. Solutions to the equations then yield the expressions,


(20) $$m_{1}=V_{o}\,c_{a}\,\text{and}\,m_{2}=K_{1}\,\int_{0}^{T}c_{a}\,d\,t-k_{2}^{\text{ROI}}\,\int_{0}^{T}m_{2}\,d\,t$$

where the ratio *m*_1_/(*m*_1_ + *m*_2_) approaches zero when *c*_*a*_ of the precursor pool in the circulation declines toward negligible levels, and the bound tracer *m*_2_ rises to represent the majority of the tracer in the brain,


(21) $$m\,\left(T\right)=K_{1}\,\int_{0}^{T}C_{a}\,\left(t\right)\,d\,t-k_{2}^{\text{ROI}}\,\int_{0}^{T}m\,\left(t\right)\,d\,t$$

where *m*(T) = *m*_1_(T) + *m*_2_(T) defines the evolutions of the three time-dependent variables,


$$K_{\text{app}}\,\left(T\right)\equiv \frac{m\,\left(T\right)}{\int_{0}^{T}C_{a}\,\left(t\right)\,d\,t}\;V_{\text{app}}\,\left(T\right)\equiv \frac{\int_{0}^{T}m\,\left(t\right)\,d\,t}{\int_{0}^{T}C_{a}\,\left(T\right)\,d\,t}$$

(22) $$\theta \,\left(T\right)\equiv \int_{0}^{T}\frac{m\,\left(t\right)\,d\,t}{m\,\left(T\right)}$$

that represent apparent clearance, apparent volume of distribution, and apparent duration of exposure, respectively. We used the definitions to obtain the six plots from different combinations of the main parameters, shown in Figures 4A–F, including the combination labeled as P2, also known as the Logan plot from arterial concentrations (Logan et al., 1990).

### Transient receptor equilibrium of bolus ligand

**FIGURE 5:**
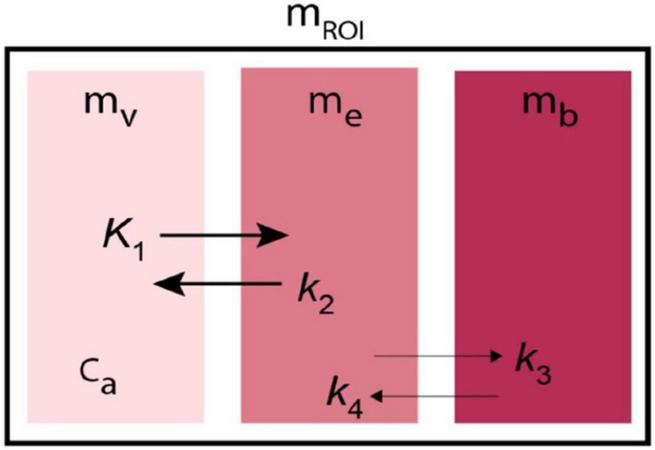
Kinetic model of Transient Receptor EquilibriuM BoLus Estimation (TREMBLE). Tracer undergoes transfer from plasma compartment (*m*_*v*_) to the exchangeable tissue compartment (*m*_*e*_) and from there to the separate compartment of specifically bound tracer (*m*_*b*_).


The method of Transient Receptor EquilibriuM BoLus Estimation (TREMBLE), first published by Sølling et al. (1997), has the advantage of identifying the precise but singular time(s) when d*B*/dt = 0. This unique method allows the users of TREMBLE to identify the quantity of ligand bound to receptors at the time of equilibrium of the quantity of bound tracer (Sølling et al., 1997; Wong et al., 1998). As illustrated in Figure 5, the method of TREMBLE uses a kinetic model of two simultaneous dynamics that include the movement of the radiotracer from the arterial compartment in plasma (*m*_*v*_) to the exchangeable brain tissue compartment (*m*_*e*_) and the transfer by binding to the additional compartment of specifically bound tracer (*m*_*b*_). A first-order differential equation describes the distribution in the vascular volume,

(23) $$\frac{d\,m_{v}\,\left(t\right)}{d\,t}=V_{0}\,\frac{d\,c_{a}\,\left(t\right)}{d\,t}$$

where *V*_0_ denotes the blood compartment volume, and *c*_*a*_(*t*) is the variable concentration in arterial plasma as a function of time. The exchange from plasma to the exchangeable compartment then obeys the equation,


(24) $$\frac{d\,m_{e}\,\left(t\right)}{d\,t}=K_{1}\,c_{a}\,\left(t\right)-\left(k_{2}+k_{3}\right)\,m_{e}\,\left(t\right)+k_{4}\,m_{b}\,\left(t\right)$$

where *K*_1_ denotes the plasma clearance, *k*_2_ the rate of exchange from tissue to plasma, and *m*_*e*_ and *m*_*b*_ denote the respective quantities in the exchangeable and specifically binding compartments,


(25) $$\frac{d\,m_{b}\,\left(t\right)}{d\,t}=k_{3}\,m_{e}\,\left(t\right)-k_{4}\,m_{b}\,\left(t\right)$$

#### Exchange compartment (m_e_)

To avoid the uncertainty of many variables, the fundamental aim of the TREMBLE analysis is to identify the quantity in the first tissue compartment *m*_*e*_ that controls the exchange of ligands with subsequent compartments. Only in the initial phase of a tomography session is the amount of radioligand bound to neuroreceptors negligible, and only then is the flux of ligands determined by the concentration difference between plasma and brain tissue.

#### Binding compartment (m_b_)

At all times, the measured radioactivity in a brain region of interest (*m*_*ROI*_) obeys the equation,


(26) $$\frac{d\,m_{\text{ROI}}}{d\,t}=V_{0}\,\frac{d\,c_{a}\,\left(t\right)}{d\,t}+K_{1}\,c_{a}\,\left(t\right)-k_{2}\,m_{e}\,\left(t\right)$$

**FIGURE 6:**
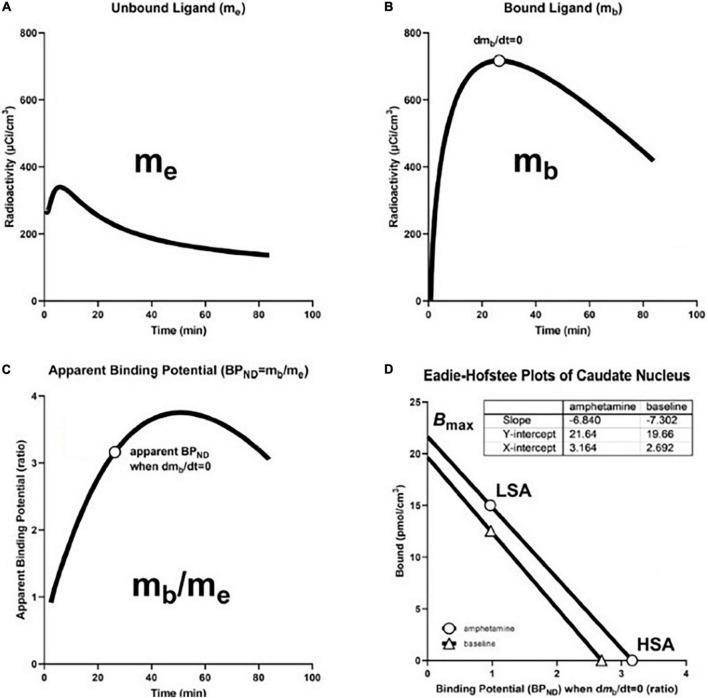
Transient Receptor EquilibriuM BoLus Estimation (TREMBLE): From precursor pool to maximum binding capacity. **(A)** Shows model-derived quantity of unbound tracer in precursor pool (*m*_*e*_) in caudate nucleus of brain of human volunteer participant in study of amphetamine effects on receptor binding of [^11^C]raclopride. **(B)** Shows model-derived quantity of specifically bound radioligand (*m*_*b*_) Circle indicates time of transient equilibrium of binding. **(C)** Shows ratio of *m*_*b*_ and *m*_*e*_ as measure of apparent binding potential (*BP*_*ND*_). Circle shows true binding potential at time of transient equilibrium of binding. **(D)** shows Eadie-Hofstee plots of binding at baseline and after amphetamine administration at high and low specific activities of [^11^C]raclopride that yield estimates of half-saturation quantities and maximum binding capacities in the absence and presence of amphetamine. Based on Sølling et al. (1997) and adapted from Wong et al. (2008).


Rearrangement of Equation 26 yields the quantity of ligand in the exchangeable compartment,


(27) $$m_{e}\,\left(t\right)=V_{e}\,\left[c_{a}\,\left(t\right)-\frac{1}{K_{1}}\,\left(\frac{d\,m_{\text{ROI}}\,\left(t\right)}{d\,t}-V_{0}\,\frac{d\,c_{a}\,\left(t\right)}{d\,t}\right)\right]$$

where *V*_*e*_ is the partition volume that equals *K_1_/k_2_*. Equation 27 is the basis of the estimation of *V_0_, K_1_*, and the derivatives of *c_*a*_(t)* and *m*_*ROI*_*(t)*. When the quantity of ligand in the exchangeable compartment is known, the bound quantity is obtained by subtraction,


(28) $$m_{b}\,\left(t\right)=m_{\text{ROI}}\,\left(t\right)-m_{v}\,\left(t\right)-m_{e}\,\left(t\right)$$

where at transient equilibrium, the first derivative of the bound quantity, *dm*_*b*_*(t)/dt*, equals zero, as shown in Figure 6. The time of this so-called transient equilibrium corresponds to a peak (or trough) of the curve of bound ligand, and this time yields the steady-state binding potential defined as the ratio of the quantity of bound ligand and the variable quantity of free ligand, here indicated by the upper case symbols, *M*_*b*_ and *M_*e*_*,

(29) $$B\,P_{\text{ND}\,\left(\text{TREMBLE}\right)}=\frac{M_{b}}{M_{e}}$$

#### Reference compartment (m_REF_)

Estimation of *V*_*e*_ (also known as V_*ND*_) requires a reference region with no binding. Assuming that the volume of precursor distribution depends on the rate constants *K*_1_ and *k*_2_ and is identical in regions of specific binding and regions of no binding, we obtained the ratio of *K*_1_ to *k*_2_ from the radioactivity in plasma and cerebellum. When the specific binding of the ligand is negligible in the cerebellum, as assumed, the kinetic model of three compartments is reduced to two compartments,


$$\frac{d\,m_{\text{REF}}}{d\,t}=\frac{d\,m_{v}\,\left(t\right)}{d\,t}+\frac{d\,m_{e}\,\left(t\right)}{d\,t}$$

(30) $$=V_{0}\,\frac{d\,c_{a}\,\left(t\right)}{d\,t}+\left(K_{1}\,c_{a}\,\left(t\right)-k_{2}^{\text{REF}}\,m_{e}\,\left(t\right)\right)$$

where $k_{2}^{\text{REF}}$refers to the magnitude of *k*_2_ in a chosen reference region such as the cerebellum. The total quantity of ligand in the reference region, *m*_*REF*_, is obtained by integration of the differential Equation 30,


(31) $$m_{\text{REF}}\,\left(T\right)=V_{0}\,C_{a}\,\left(T\right)+K_{1}\,\int_{0}^{T}c_{a}\,\left(t\right)\,d\,t-k_{2}^{\text{REF}}\,\int_{0}^{T}m_{e}\,\left(t\right)\,d\,t$$

where *m*_*e*_ is expressed in terms of *m*_*REF*_ because *m*_*e*_ is unknown. Substitution of *m*_*e*_ with the expression *m*_*e*_ = *m*_*REF*_ (1 *- m_*v*_)/m*_*REF*_ yields,


$$m_{\text{REF}}\,\left(T\right)=V_{0}\,C_{a}\,\left(T\right)+K_{1}\,\int_{0}^{T}c_{a}\,\left(t\right)\,d\,t-k_{2}^{\text{REF}}$$

(32) $$\int_{0}^{T}m_{\text{REF}}\,\left(t\right)\,\left(1-\frac{m_{v}\,\left(t\right)}{m_{\text{REF}}\,\left(t\right)}\right)\,d\,t$$

where, at later times, the approaching equilibrium state is associated with substantial washout of the ligand from plasma as it accumulates in the brain and other organs. When the magnitudes of *c_*a*_(t)* and the ratio m_*v*_/m_*REF*_ become negligible, Equation (32) reduces to,


(33) $$m_{\text{REF}}\,\left(T\right)=K_{1}\,\int_{0}^{T}c_{a}\,\left(t\right)\,d\,t-k_{2}^{\text{REF}}\,\int_{0}^{T}m_{\text{REF}}\,\left(t\right)\,d\,t$$

where division of Equation (33) by the integral of the arterial concentration yields a simple linearized solution as previously described by Gjedde (1982),


(34) $$\frac{m_{\text{REF}}\,\left(T\right)}{\int_{0}^{T}c_{a}\,\left(t\right)\,d\,t}=K_{1}+k_{2}^{\text{REF}}\,\frac{\int_{0}^{T}m_{\text{REF}}\,\left(t\right)\,d\,t}{\int_{0}^{T}c_{a}\,\left(t\right)\,d\,t}$$

where the cerebellar rate constants *K*_*1*_ and $k_{2}^{\text{REF}}$ are solved graphically from the y-intercept and slope, respectively, and *V*_*e*_ is the ratio of the reference region *K*_*1*_ and $k_{2}^{\text{REF}}$.

#### Vascular volume (*V*_*o*_)

It is possible to estimate regional *V*_0_ and *K*_1_ values by means of the single-tissue compartment model applied to the estimation of mROI. To obtain the estimate of *K*_*1*_, it is possible to focus on the onset of acquisition of the PET images when the tracer predominantly exists in the vascular volume, and the loss of tracer from the brain to plasma can be ignored,


(35) $$m_{\text{ROI}}\,\left(T\right)=V_{0}\,c_{a}\,\left(T\right)+K_{1}\,\int_{0}^{T}c_{a}\,\left(t\right)\,d\,t$$

where *m*_*ROI*_*(T)* is the radioactivity in the region of interest. When divided by *c_*a*_(t)*, Equation (35) yields the equation of the GP-SI plot (also see Equation 7),


(36) $$\frac{m_{\text{ROI}}\,\left(T\right)}{c_{a}\,\left(t\right)}=V_{0}+K_{1}\,\frac{\int_{0}^{T}c_{a}\,\left(t\right)\,d\,t}{c_{a}\,\left(t\right)}$$

where the value of *V*_0_ is measured from the ordinate axis and the value of *K*_*1*_ is obtained from the initial slope of the uptake obtained by the initial acquisition frames, for example, at 0–2 min post-injection.

### Alternative transient equilibrium method

The ligand and neuroreceptor interaction described by Farde et al. (1988, 1989) involves a three-compartment model with four first-order rate constants shown in Figure 5. In TREMBLE, the center compartment of a region of interest represents the quantity of exchangeable ligand. In contrast, in the Transient Equilibrium Method (TEM), the center compartment of a region of interest represents the unbound or free quantity of the tracer in a region of reference, denoted as *m*_*f*_. The exchange of ligand in the compartments then can be described by two differential equations,


(37) $$\frac{d\,m_{f}\,\left(t\right)}{d\,t}=K_{1}\,c_{a}\,\left(t\right)-\left(k_{2}+k_{3}\right)\,m_{f}\,\left(t\right)+k_{4}\,m_{b}^{*}\,\left(t\right)$$

and


(38) $$\frac{d\,m_{b}\,\left(t\right)}{d\,t}=k_{3}\,m_{f}\,\left(t\right)-k_{4}\,m_{b}^{*}\,\left(t\right)$$

where *m*_*f*_ denotes the free tissue quantity and *c*_*a*_ is the plasma concentration of the radiotracer. For convenience, the specifically bound quantity in tissue alleged by TEM is denoted with an asterisk as $m_{b}^{*}$. Under the assumption that the specific binding of [^11^C]raclopride is negligible in the reference region, the exchange of ligand in the reference region is reduced to,


(39) $$\frac{d\,m_{\text{REF}}\,\left(t\right)}{d\,t}=K_{1}\,c_{a}\,\left(t\right)-k_{2}\,m_{f}\,\left(t\right)$$

and


(40) $$d\,m_{\text{ROI}}\,\left(t\right)=d\,m_{\text{REF}}\,\left(t\right)+d\,m_{b}^{*}\,\left(t\right)$$

where by rearrangement of Equation 40, the specifically bound quantity yields,


(41) $$m_{b}^{*}\,\left(t\right)=m_{\text{ROI}}\,\left(t\right)-m_{\text{REF}}\,\left(t\right)$$

such that the alleged specifically bound quantity $m_{b}^{*}$ is obtained with TEM by subtraction of the quantity in the reference region *m*_*REF*_ from the total quantity *m*_*ROI*_. In this calculation, it is assumed that the free quantities in tissue and plasma have equal magnitudes in *m*_*REF*_ and *m*_*ROI*_. As in TREMBLE, the specifically bound quantity then is obtained in TEM at the time of transient equilibrium when the first derivative $d\,m_{b}^{*}\,\left(t\right)\,d\,t$ equals zero. The equilibrium binding potential in TEM is measured from the bound quantity in a region of binding divided by the total quantity of tracer in the reference region,


(42) $$B\,P_{N\,D\,\left(T\,E\,M\right)}=\frac{M_{b}^{*}}{M_{\text{REF}}}$$

where it is important to note that the binding potential obtained by TREMBLE in principle, therefore, is different from the potential obtained by TEM because the assumed non-displaceable quantities have different origins and are determined differently.

### From binding potential to receptor density

We determined receptor density and affinity graphically by means of the Eadie-Hofstee plot shown in Figure 6D, based on binding values obtained at two different degrees of receptor occupancy. We used the Eadie–Hofstee plot rendition of the linear relationship between the steady-state estimates of *BP*_*ND*_ as functions of the bound quantity (*B*) of ligand (Figure 6D),


(43) $$B=B_{\text{max}}-\left(V_{d}\,K_{D}\right)\,B\,P_{\text{ND}},$$

where the ordinate intercept indicates the value of the receptor density *B*_max_, and the slope represents the value of the half-saturation concentration, as related to the reciprocal of the affinity constant *1/K_*D*_*.

## Modeling brain receptor binding by regional reference region

### Distribution volume ratio from regions of interest and reference

We obtained graphical plots of the Distribution Volume Ratio (DVR) equal to the value of *BP*_*ND*_ + 1. The distribution volume ratio derived from a reference value from an assumed reference region yielded a reparameterized reference region mass originally derived from arterial concentrations. With the approach of steady-state, the slope of the DVR with respect to the normalized integral of the reference region is the value of the binding potential *BP*_*ND*_. The ordinate intercept is 1/$k_{2}^{\text{REF}}$ where the target tissue ROI and the reference region REF (e.g., cerebellum for [^11^C]raclopride) replace integral time Θ (defined below by Equation 47). In the presence of a reference region, we define the tracer mass in this region as *M*_*REF*_ and derive the term for DVR (Gjedde, 2003),


(44) $$M_{\text{REF}}\,\left(T\right)=K_{1}\,\int_{0}^{T}c_{a}\,\left(t\right)\,d\,t-k_{2}\,\int_{0}^{T}M_{\text{REF}}\,\left(T\right)\,d\,t$$

(45) $$\int_{0}^{T}c_{a}\,\left(t\right)\,d\,t=\frac{M_{\text{REF}}\,\left(T\right)}{K_{1}}+\frac{k_{2}}{K_{1}}\,\int_{0}^{T}M_{\text{REF}}\,\left(t\right)\,d\,t$$

(46) $$\int_{0}^{u}\int_{0}^{T}c_{a}\,\left(t\right)\,d\,t\,d\,T=\frac{\int_{0}^{u}M_{\text{REF}}\,\left(t\right)\,d\,T}{K_{1}}+\frac{k_{2}}{K_{1}}\,\int_{0}^{u}\int_{0}^{T}M_{\text{REF}}\,\left(t\right)\,d\,t\,d\,T$$

(47) $$\theta \,\left(T\right)=\left[\frac{\int_{0}^{T}M_{\text{REF}}\,d\,t}{M_{\text{REF}}}\right]$$

(48) $$\theta^{\prime }\,\left(T\right)=\left[\frac{\int_{0}^{T}\int_{0}^{u}M_{\text{REF}}\,d\,t\,d\,u}{\int_{0}^{T}M_{\text{REF}}\,d\,t}\right]$$

(49) $$\text{DVR}\,\left(T\right)=\frac{\int_{0}^{T}M_{\text{ROI}}\,\left(T\right)\,d\,t}{\int_{0}^{T}M_{\text{REF}}\,\left(T\right)\,d\,t}$$

where Equations 44–49 lead to the formulation of the distribution volume ratio (DVR) in terms of measured quantities in a brain tissue region of interest because of binding (ROI) and the chosen reference region of absent specific binding (Logan et al., 1996),


$$\text{DVR}\,\left(T\right)=\frac{\int_{0}^{T}M_{\text{ROI}}\,\left(T\right)\,d\,t}{\int_{0}^{T}M_{\text{REF}}\,\left(T\right)\,d\,t}=\left(\frac{k_{2}\,k_{3}}{k_{2}+k_{3}}\right)\,\theta^{\prime }\,\left(T\right)$$

(50) $$+R_{1}\,\left(\frac{k_{2}\,k_{3}^{\text{ROI}}}{k_{2}^{\text{REF}}\,k_{3}^{\text{ROI}}}\right)\,\left[1-\frac{\left(\frac{M_{\text{ROI}}\,\left(T\right)}{R_{1}\,M_{\text{REF}}\,\left(T\right)}-1\right)}{\left(k_{2}+k_{3}^{\text{ROI}}\right)\,\left(T\right)}\right]$$

where *R*_1_ is the ratio of the initial clearances of the tracer into a binding region of interest (ROI) and the reference region (REF) of no binding. The resulting equation now applies to the reference region version of the equation suitable for analysis of irreversible or reversible binding to receptors,


$$\text{DVR}\,\left(T\right)=\left(\frac{k_{2}\,k_{3}}{k_{2}+k_{3}}\right)\,\theta^{\prime }\,\left(T\right)-\frac{R_{1}\,k_{2}\,k_{3}^{\text{ROI}}}{{\left(k_{2}^{\text{REF}}+k_{3}^{\text{ROI}}\right)}^{2}}$$

$$\;+\frac{R_{1}}{k_{2}^{\text{REF}}+k_{3}^{\text{ROI}}}\,\left[k_{2}+k_{3}^{\text{ROI}}-k_{2}^{\text{REF}}\,\left(\frac{k_{2}}{k_{2}^{\text{REF}}+k_{3}^{\text{ROI}}}-1\right)\,e^{-\alpha \,\theta^{\prime }\,\left(T\right)}\right]$$

### Slope-intercept plot application to reference region approach

**FIGURE 7:**
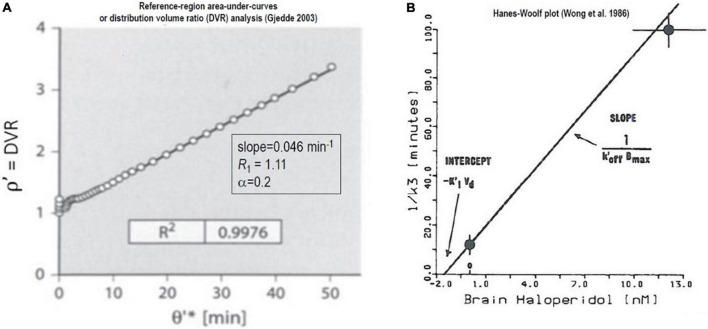
Non-saturable binding relative to reference region: Irreversible binding of [^11^C]NMSP. Graphical plots of an irreversibly binding radiotracer with a reference region. **(A)** Shows Distribution Volume Ratio (DVR) relative to cerebellum of [^11^C]NMSP binding in human striatum vs. time variable θ′ (Gjedde, 2003). **(B)** Hanes-Woolf plot of time constants of binding as function of inhibitor concentration (Wong et al., 1986a,b, c).

**FIGURE 8:**
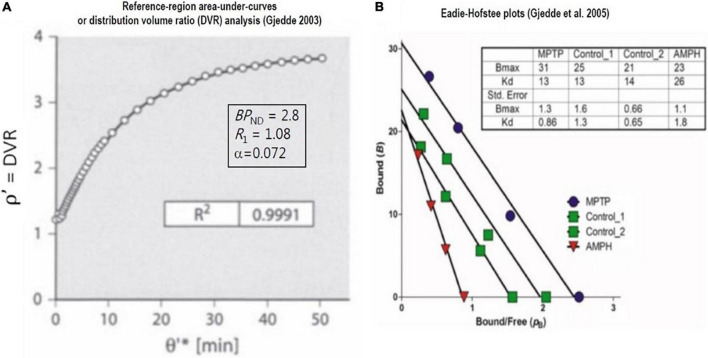
Saturable binding relative to reference region: Reversible binding of [^11^C]raclopride. Graphical plots of irreversibly binding radiotracer with reference region. **(A)** Shows plot of distribution volume ratio (DVR) of reversible radioligand vs. double integral of reference region in single subject (Gjedde, 2003). **(B)** Shows Eadie-Hofstee plots of bound ligand (*B*) vs. binding potential (*p*_*B*_ or *BP*_*ND*_) of [^11^C]raclopride in monkeys post-MPTP compared to controls, yielding estimates of receptor density (*B*_max_) and half-saturation mass (*K’*_*D*_), reported by Holden et al. (2002) and adapted by Gjedde et al. (2005).


In Figures 7 and 8, we present plots of the DVR measure vs. the double integral of normalized time (see Equation 48). The time variable θ′ equals the twice integrated tracer mass in the reference region relative to the once integrated tracer mass integral, and *R*_*1*_ is the clearance (*K*_*1*_) by a region of interest (ROI), divided by the reference region (e.g., cerebellum),

(52) $$D\,V\,R=1+k_{3}\,\theta^{\prime }\,\left(T\right)-\left(1-R_{1}\right)\,e^{-\alpha \,\theta^{\prime }\,\left(T\right)}$$

where in the following Equation (53), ρ* is the integral of the distribution volume ratio (DVR), and *p*_*B*_ is the binding potential (*BP*_*ND*_) when net binding (*k*_3_) is zero at an apparent steady-state of binding,


(53) ρ*=1+pB⁢(1-e-θ*′)-(1-R1)⁢e-α⁢θ*′

or, with the alternative nomenclature introduced by Innis et al. (2007),


(54) $$\text{DVR}=1+B\,P_{\text{ND}}\,\left(1-e^{-\alpha \,\theta^{\prime }\,\left(T\right)}\right)-\left(1-R_{1}\right)\,e^{-\alpha \,\theta^{\prime }\,\left(T\right)}$$

where the equation for *R*_1_ = 1 is:


(55) $$\text{DVR}=1+B\,P_{N\,D}\,\left(1-e^{-\alpha \,\theta^{\prime }\,\left(T\right)}\right)$$

where sufficiently extended times (*T*) yield the common basis for interpretation of *DVR*,


(56) $$\text{DVR}=1+B\,P_{N\,D}$$

### Irreversible binding of [^11^C] NMSP relative to reference region

As an illustration of the graphical plot of the binding of the irreversibly binding radiotracer [^11^C]NMSP with a reference region, Figure 7A shows the graphical analysis of the DVR measure of [^11^C]NMSP vs. the time variable θ′*(T)*,


$$\text{DVR}\,\left(T\right)=\left(\frac{k_{2}\,k_{3}}{k_{2}+k_{3}}\right)\,\theta^{\prime }\,\left(T\right)$$

$$+R_{1}\,\left[\frac{k_{3}}{k_{2}+k_{3}}+\left(\frac{k_{2}}{k_{2}+k_{3}}\right)\,e^{-\alpha \,\theta^{\prime }\,\left(T\right)}\right]$$

(57) $$+{\left(\frac{k_{2}}{k_{2}+k_{3}}\right)}^{2}\,\left[1-e^{-\alpha \,\theta^{\prime }\,\left(T\right)}\right]$$

The maximum binding capacity *B*_max_ calculated from the effect of the unlabeled inhibitor (haloperidol) on the time constant of [^11^C]NMSP binding by the Woolf (or Hanes-Woolf) Plot of Figure 7B, using the equation adopted by Wong et al. (1986b),


(58) $$\frac{1}{k_{3}}=\frac{K_{\text{I}}\,V_{d}+M_{\text{I}}}{B_{\text{max}}\,k_{\text{off}}^{\prime }}$$

where the symbols have their usual meaning, including half-saturation mass (*K*_I_*V*_*d*_) and mass of the inhibitor (*M*_I_), the maximum quantity of receptors (*B*_max_), and the inhibitor off-rate at the receptors$(k_{\text{off}}^{\prime }$).

### Reversible binding of [^11^C]raclopride relative to reference region

When derived from the equations above, the graphical method also applies to reversible radioligands with a reference region. Here, we illustrate the application to the reversible binding of the radioligand [^11^C]raclopride. As we show in Figure 8A, the plot often is equivalent to the so-called “non-invasive” version of the Logan plot that adopts the records of the tracer in regions of interest and a region of reference,


(59) $$\frac{\int_{0}^{\text{T}}\left(m_{\text{ROI}}-m_{\text{REF}}\right)\,\text{dt}}{m_{\text{ROI}}}=B\,P_{\text{ND}}\,\frac{\int_{0}^{\text{T}}m_{\text{REF}}\,\text{dt}}{m_{\text{ROI}}}-\frac{\left(1-{\text{e}}^{-\alpha \,t}\right)}{k_{2}^{\text{REF}}}$$

where Figure 8B is the Eadie–Hofstee plot that provides estimates of dopamine receptor density and radioligand affinity. The plot was applied to recordings of [^11^C]raclopride binding in brains of monkeys having a parkinsonian condition after exposure to methyl-phenyl-tetra-hydro-pyridine (MPTP) and to control animals without this condition (Evans et al., 1992; Doudet et al., 1993). As explained above, the Eadie–Hofstee plots are linearizations of the fundamental relationship that defines the variable *BP*_*ND*_.

### Reversible binding relative to reference region by transient equilibrium method

**FIGURE 9:**
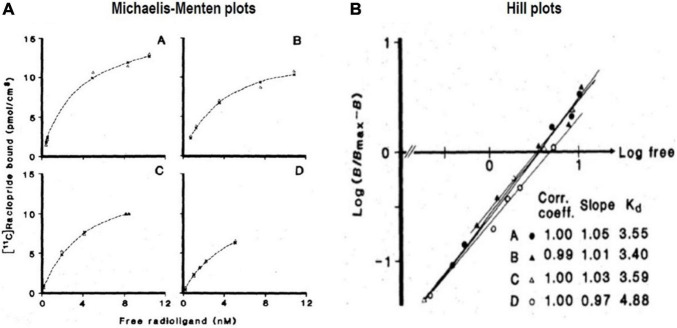
Transient Equilibrium Method (TEM): From bound tracer to maximum binding capacity. **(A)** Shows Michaelis and Menten (1913) plots of bound quantities of tracer [^11^C]raclopride vs. free radioligand concentrations (nM) of four healthy subjects (A–D) in human putamen). **(B)** Shows Hill plots of half-saturation concentration (*K*_D_) determination from striatal binding of [^11^C]raclopride administered to the four subjects, yielding *K*_D_ values from intersection with abscissa and *B*_max_ values obtained from analysis shown in **(A)** (adapted from Farde et al., 1986).

Farde et al. (1986) estimated the binding of [^11^C]raclopride in the striatum by a semi-graphical approach to the *B/F* ratios calculated from the peak of the resulting values of the [*M*(T)/*M*_*e*_(T)]-1 quantity for which the authors assumed d*B*/dt = 0. The authors adopted the approach on the assumption that the unbound quantity of tracer can be determined in a non-binding region of the brain, such as the cerebellum, assumed to be without dopamine receptors, as illustrated for the four subjects A–D of Figures 9A,B. When carried out with progressively unlabeled mass injections of raclopride in the presence of the radiotracer, as many as five measurements of B/F yielded the estimates of *B*_max_ of Figure 8A and the estimates of *K*_D_ shown here by the Hill plots of Figure 8B. While the transient equilibrium method (TEM) approach has been widely used, it has disadvantages that include the uncertainty of the precise time and magnitude of d*B*/dt = 0, as elaborated above in the section on TREMBLE. Alternatively, an unlabeled but chemically different inhibitor serves to estimate different varying total volumes of distribution (*V*_*T*_) of the tracer as elaborated in the outline of Inhibition plots below. The approach requires a procedure that yield the distribution volume of non-displaceable (V_*e*_ or *V*_*ND*_) tracer in the brain tissue.

### Linearized plots of reversible binding relative to reference region

**FIGURE 10:**
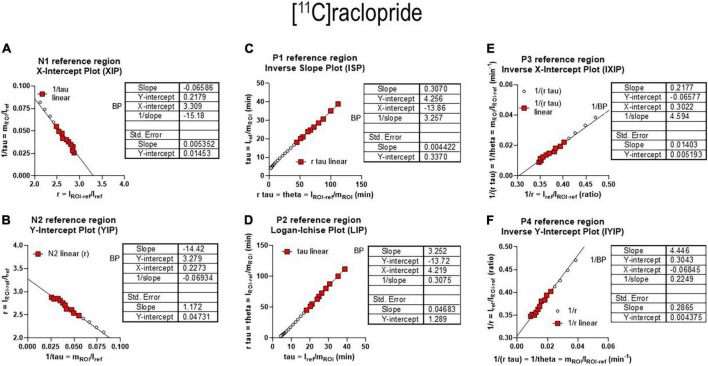
Receptor binding relative to non-binding reference region. **(A)** Left graph reference region N1 graphical plot. **(B)** Right graph reference N2 graphical plot. **(C)** Left graph reference region P1 plot. **(D)** Right graph reference region P2 plot, equivalent to Logan reference region plot. **(E)** Left graph reference region P3 plot. **(F)** Right graph reference region P4 plot (adapted from Gjedde, 2003 and Zhou et al., 2003).

The six plots of Figure 10 are the reference regions variants of the six plots of graphical analysis presented above for estimates based on arterial concentration measurements. The plots are characterized by an approach to linear relations of the dependent and independent variables after reversible binding of the radiotracer (i.e., *k*_4_ > 0). Each of the plots has a positive or negative slope for the designated P1–P4 and N1–N2 graphs, respectively, depending on the sign of the slope. Each of the panels of Figure 10 shows the formula for the calculation of the primary outcome measures for each of the lines. Thus, the P1 plot is a graph of the equation Y = [X/*BP*_*ND*_] + [1/(*k*_2_’ *BP*_*ND*_)] where Y (tau) is the integral of *m*_*REF*_ relative to *m*_*ROI*_ with unit of minutes while X (theta) is the integral of the difference between *m*_*ROI*_ and *m*_*REF*_ relative to *m*_*ROI*_ with unit of minutes.

## Modeling brain receptor binding without reference region

### Binding potential determination by inhibition without reference region

Neuroreceptor studies of the brain *in vivo* with PET normally are possible only by comparing the experimentally determined values of binding potentials (*BP*_*ND*_) of receptor radioligands at multiple distinguishable levels of receptor inhibition in order to yield receptor density and affinity estimates. In some cases, however, there are radioligands for which a true reference tissue of only non-specific binding of the radioligand is not available. In these cases, we evaluated three linearizations of the basic receptor availability equation derived for estimating the magnitude of the reference distribution volume of the radioligand in a region of non-displaceable binding by simple linear regression. Here, the symbol *V*_*ND*_ (or V_*e*_) refers to the volume of distribution of the non-displaceable radiotracer in a compartment with no specific binding, also known as the partition coefficient or volume of the radioligand.

To estimate *B*_max_ and *K*_*I*_ in brain tissue in the absence of a non-specifically binding region, it is necessary to estimate the inhibition of the radioligand binding at the receptor of interest. In the test presented in the example below, the receptor of interest is the α7 nicotinic acetylcholine receptor (nAChR), for which the total volumes of distribution (V(T) or *V*_*T*_) were determined at baseline and after different degrees of occupation by an inhibitor. With no known non-specifically binding (reference) region, it is possible to estimate the unknown values of *V*_*ND*_ (V_*e*_) from the graphical plots of values of *V*_*T*_ (V(T)) at baseline and different degrees of occupancy. The theoretical value of *V*_*ND*_ emerged from an extrapolation of the data to an intercept with one of the axes or to the line of identity of pairs of values of *V*_*T*_ at baseline and different degrees of occupancy.

In these cases, three linearizations in the shape of graphical plots emerged when the authors of publications by Lassen et al. (1995); Gjedde and Wong (2001), and Cunningham et al. (2010) linearized the basic equation of receptor availability in three different ways, identified here as the Saturation, Inhibition, and Occupancy plots, to avoid the uncertainty of the Lassen Plot that no longer refers exclusively to the actual plot that Lassen et al. (1995) derived and reported in 1995.

We first linearized the fractional receptor availability expressed by Equation (19) into three separate equations. All three linearizations yielded the reference tissue volume of distribution (V_*e*_ or *V*_*ND*_) required to estimate the binding potential (*BP*_ND_) of a radiotracer. The quantification of *BP*_ND_ uses separate estimates of radiotracer distribution volumes of a given radiotracer to obtain a value for the variable now generally known as receptor availability (Kety and Schmidt, 1948; Raichle et al., 1983; Rescigno and Beck, 1987; Ohta et al., 1996; Gjedde, 2008),


(60) $$a=1-s=\frac{V_{\text{T}\,\left(\text{i}\right)}-V_{\text{ND}}}{V_{\text{T}\,\left(\text{b}\right)}-V_{\text{ND}}}$$

that is an estimate of the fractional number of receptors available for binding by the radioligand (1 – *s*) in terms of the relevant volumes of distribution when the term *s* represents the occupancy of the ligand. The volume of distribution terms represent baseline and inhibition states of the radioligand, including the values of the total volumes of distribution (*V*_*T*_). Of the terms, *V*_*T(i)*_ reflects the degree of inhibition by binding, i.e., the apparent total volume of distribution of the sum of the specifically bound and non-specifically dissolved ligand molecules occupying the receptor. The term *V*_*T(b)*_ now reflects the baseline state of the apparent total volume of distribution that is characteristic of the situation that prevails when the neuroreceptor is unoccupied by an unlabeled inhibitor.

**FIGURE 11:**
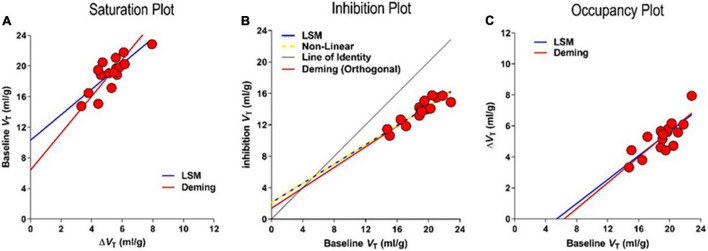
Competition of tracer [^18^F]ASEM with unlabeled inhibitors. Results of regressions to three different linearizations of competition plots of effect of unlabeled drug interaction with PET tracer binding in absence of valid reference region. **(A)** Saturation Plot of baseline *V*_*T*_ vs. change of *V*_*T*_ with inhibition. **(B)** Inhibition Plot of baseline vs. inhibition *V*_*T*_ yielding saturation *s* as 0.39 ± 0.077 and non-displaceable volume *V*_*ND*_ as 5.4 ± 2.75 ml/gm **(C)** Occupancy Plot of change of *V*_*T*_ vs. baseline *V*_*T*_ (adapted from Horti et al., 2014; Khodaii et al., 2022).

We used the three linearly transformed plots to obtain estimates of *BP*_*ND*_ that complete the primary analysis of binding. The term *BP*_*ND*_ also enters into the Eadie-Hofstee Plot, which is the linear form of the Michaelis-Menten Equation that yields both the maximum binding (*B*_max_) and the affinity coefficient (half-saturation content or mass), *K*_D_*V*_*d*_, of the neuroreceptors (also see Equations 14, 43, and 58),


(61) $$B=B_{\text{max}}-K_{\text{D}}\,V_{d}\,B\,P_{\text{ND}}$$

where *B* is the mass of bound radioligand. The *BP*_*ND*_ is defined as the ratio of distribution volumes of displaceable and specifically bound tracer, divided by the non-displaceably bound (non-specific) radioligand masses (Sokoloff et al., 1977; Gjedde, 1982). To determine the *BP*_*ND*_ of a radioligand, the distribution volumes are entered into the equation defining the term *BP*_*ND*_ (Rescigno and Beck, 1987; Kuikka et al., 1991; Gjedde et al., 2005),


(62) $$B\,P_{\text{ND}}=\frac{V_{\text{T}}-V_{\text{ND}}}{V_{\text{ND}}}$$

which applies both to the receptor binding baseline and to multiple degrees of receptor blockade, provided the *V*_*ND*_ estimate is unaffected by the blockade. From the volumes of distribution of the radioligand in the absence and presence of displaceable binding in a baseline state and states of inhibition of the receptors, the three linearizations yield the values of *BP*_*ND*_ of radioligands shown in Figures 11A–C, (Horti et al., 2014). The three linearizations often appear under the heading of “Lassen” plots in honor of the publication of the first of the three plots in 1995 but here follow the separate terms of the Saturation, Inhibition, and Occupancy plots.

#### Saturation plot

As a novel, steady-state approach to determining *BP*_*ND*_ of tracers with an unknown reference volume of distribution, Lassen et al. (1995) compared two values of receptor occupancy, one at only negligible labeled tracer concentration and one in the mid-range of occupancy, by addition of unlabeled ligand. The unlabeled radioligand concentration in brain tissue water would be close to zero in the radiotracer-only case, with an unchanged concentration at the subsequent inhibition. To estimate the non-specific binding volume, Lassen et al. (1995) solved and linearized Equation (60) to obtain the Saturation Plot. The plot yields the measures of *V*_*ND*_ by plotting on the ordinate the distribution volume at baseline (*V*_*T(b*)_) as a function of the difference between the baseline and inhibition (*V*_*T(i*)_) distribution volumes, respectively, plotted on the abscissa as,


(63) $$\Delta \,V_{\text{T}}=V_{\text{T}\,\left(\text{b}\right)}-V_{\text{T}\,\left(\text{i}\right)}$$

that yields the plot shown in Figure 11A,


(64) $$V_{\text{T}\,\left(\text{b}\right)}=\frac{1}{s}\,\Delta \,V_{\text{T}}+V_{\text{ND}}$$

where the estimate of *V*_*ND*_ is the ordinate intercept, and the estimate of the ratio 1/s is the slope of the regression.

#### Inhibition plot

We independently solved and linearized Equation (60) to obtain the Inhibition Plot (Gjedde and Wong, 2001). The plot yields estimates of *V*_*ND*_ from the inhibition volume *V*_*T(i)*_ as a function of the baseline *V*_*T(b)*_, using the linear regression of Figure 11B,


(65) $$V_{T\,\left(i\right)}=\left(1-s\right)\,V_{T\,\left(b\right)}+s\,V_{N\,D}$$

where *V*_*ND*_ is the intercept of the regression line with the line of identity.

#### Occupancy plot

Cunningham et al. (2010) used the inverted axes of the Saturation Plot and demonstrated the use of graphical analysis of this relationship at each of the different doses of an unlabeled inhibitor to estimate occupancies. We define the inverted axes of the Saturation Plot as the “Occupancy Plot” to avoid the less specific “Lassen Plot” term. The linear transformation known as the Occupancy Plot yields estimates of differences between the distribution volumes at baseline and during inhibition (Δ_V*T*_) as a function of the distribution volume at baseline (see Figure 11C),


(66) $$\Delta \,V_{T}=s\,V_{T\,\left(b\right)}-s\,V_{N\,D}$$

where *V*_*ND*_ is the intercept on the abscissa. Thus the Occupancy and Saturation plots have inverted axes with respect to each other.

Based on goodness of fit parameters for two regression methods (Deming II and Least Squares regressions), the Occupancy and inhibition Plots have higher degrees of convergence, probably because of the avoidance of the mixtures of dependent and independent variables of the Saturation plot. The average differences in results of the Least Squares and Deming II applications to the Inhibition and Occupancy plot linearizations are negligible (less than 1%). In comparison, we found more than a 35% difference in the results of the Saturation plots using the Deming II and Least Squares regressions. This most likely was due to the non-negligible variability of independent variables. The three plots yield the same parameter estimates in noiseless conditions, but the Occupancy and Inhibition Plots provide similar convergences in the presence of noise (Khodaii et al., 2022), as listed in Table 2.

**TABLE 2:** Bold values with standard errors (SE) refer to regressions of which they are the primary results.

Plot	Saturation		Inhibition			Occupancy	
Regression	Linear	Deming II	Non-linear	Linear	Deming II	Linear	Deming II
*1/s* ± SE	**1.67 ± 0.33**	**2.41± 0.76**	2.56	2.56	2.86	2.56	2.38
*V*_*ND*_ ± SE	**10.3 ± 1.78**	**6.40 ± 3.80**	**5.43 ± 2.75**	5.36	3.89	5.36	6.33
*1-s* ± SE	0.40	0.59	0.61	**0.61 ± 0.08**	**0.65 ± 0.11**	0.61	0.58
*s V*_*ND*_ ± SE	6.16	2.66	2.12	**2.09 ± 1.47**	**1.36 ± 1.99**	2.09	2.66
*s* ± SE	0.60	0.41	0.39 ± 0.08	0.39	0.35	**0.39 ± 0.08**	**0.42 ± 0.11**
-*s V*_*ND*_ ± SE	−6.16	−2.66	−2.12	−2.09	−1.36	−**2.09 ± 1.47**	−**2.66 ± 2.13**

Identical colors indicate consistency across variable. Note consistency of inhibition plots.

### Binding capacity analysis in absence of reference region

We found the occupancy values of the nicotinic a7 acetylcholine receptor (a7 nAChR) in the human brain to be relatively low when investigated in the presence of the inhibitor DXMB-A (GTS-21). In primates, the values of *V*_*ND*_ by means of the Inhibition Plot revealed a much higher degree of saturation, as shown in Figure 11B (Horti et al., 2014; Wong et al., 2018).

**FIGURE 12:**
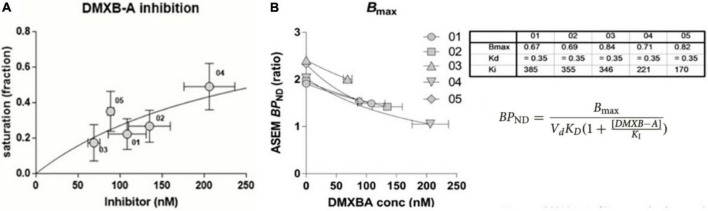
Binding parameter estimates in absence of reference region. **(A)** Estimates by [^18^F]ASEM of occupancy in humans of α7 nAChR by pairs of *BP*_*ND*_ at specific inhibitor DXMB-A concentrations. **(B)** Determination of *B*_max_ and V_*d*_ *K*_D_ by means of Equation 68, listed in table for five healthy volunteers receiving single oral dose of DXMB-A (GTS-21) (adapted from Wong et al., 2018).


The determination of the value of *V*_*ND*_ in primates by PET led to the application of two pairs of *V*_*T*_ estimates in humans by PET with [^18^F]ASEM in the absence and presence of DXMB-A (GTS-21). This allowed us to estimate the value of *BP*_*ND*_, as shown in Figure 12A. The findings lead to the calculation of values of *B*_max_ and *K*_*I*_ or *K*_D_, as shown in Figure 12B. Thus, with a true reference region, *V*_*T*_ and *BP*_*ND*_ (equal to [*V*_*T(ROI)*_/*V*_*T(REF)*_]-1) were determined for both irreversibly and reversibly binding radiotracers. Binding potentials at baseline (b) and inhibition (i) then observe the relationships,

(67) $$B\,P_{\text{ND}\,\left(\text{b}\right)}=\frac{V_{\text{T}\,\left(\text{b}\right)}}{V_{\text{ND}\,\left(\text{b}\right)}}-1\,\text{and}\,B\,P_{\text{ND}\,\left(\text{i}\right)}=\frac{V_{\text{T}\,\left(\text{i}\right)}}{{\text{V}}_{\text{ND}\,\left(\text{i}\right)}}-1$$

or,


(68) $$B\,P_{\text{ND}}=\frac{B_{\text{max}}}{V_{d}\,K_{D}\,\left(1+\frac{\left[D\,M\,X\,B-A\right]}{K_{\text{I}}}\right)}$$

where the calculations of values of *B*_max_ and V_*d*_*K*_D_ are illustrated in Figure 12B for five healthy subjects, each receiving one oral dose of DXMB-A (GTS-21).

It is often necessary to correct for a change of the reference distribution volume when plasma and potentially brain tissue free fractions (*f*_*p*_) change in response to an unlabeled inhibitor or drug challenge. Then it is necessary to consider different values of the distribution volume of a non-displaceable radioligand in a non-specifically binding region (*V*_*ND*_) for the baseline and inhibition conditions, as shown in Equation (67). The variable values of *V*_*ND*_ yield a version of the Inhibition Plot that Phan et al. (2018) described as the Extended Inhibition Plot (EIP) that considered both *f*_*p*_ and *V*_*ND*_ changes. The *V*_*ND*_ at the baseline condition (*V*_*ND(b*)_) must already be available to solve for the *V*_*ND*_ in the inhibited situation (*V*_*ND(i*)_). This situation is relevant to pharmacological drug challenges that affect the binding to the receptor by changing the release or depletion of endogenous neurotransmitters without a change in *f*_*p*_. However, a challenge with an unlabeled drug may increase *f*_*p*_ when the challenge displaces radioligand bound to plasma proteins, as shown in Figure 12B (see table insert). As an example, a challenge with unlabeled yohimbine may reduce the protein binding of the radioligand when up to 82% of radiolabeled yohimbine is bound to plasma proteins (Berlan et al., 1993).

## Discussion

### Neuroreceptor mapping *in vivo*

The purpose of kinetic neuroimaging is to raise the understanding of changes in brain functions that occur with age and the decline of these functions because of disease. By means of the methods of neuroimaging, neurokineticists acquire insights that, in the most fortuitous of cases, lead to new disease treatments in disciplines as varied as neurology, neurosurgery, neuropsychiatry, and disorders of consciousness. In the last four decades, neurokineticists took advantage of the rise of a broad spectrum of technologies and therapies that provide new opportunities for the study of brain networks with improved levels of resolution, as well as for the observation of the basic mechanisms of the brain’s kinetic work.

Live brain tomography with molecular tracers is a serious challenge for researchers. The images need quantitative interpretation. Prior to molecular imaging, brain images necessarily were anatomical and required interpretation according to the size of the tumor, the degree of medial displacement, or the massive results or signs of bleeding. Later, with functional images from comparisons of active and passive states, researchers faced the challenges of adding numerical performance indicators to the qualitative properties of functional neuroimages.

Here, we described the general principles of quantifying brain images as they relate to brain function, and we considered common analytical approaches. The approaches provided an understanding of current findings and the frequent quantification improvements that followed the increasing complexity of brain imaging methods (Gjedde et al., 2011). While the work of the brain includes the fundamental roles of blood flow and energy metabolism, neurokineticists observe that neuronal networks and neurotransmitter systems undergo changes that are equally, if not more important. The changes appear to be the results of mechanisms that neurokineticists do not understand in sufficient detail. The mechanisms include the formation of potentially pathological substances in the form of abnormal proteins in the tissues of the brain, with the resulting disruption of second messenger cascades that seem to underlie the declining brain functions among the elderly and the old.

From the introduction of the early imaging of emitted photons with radioactive gases in the 1950s to the advent of positron imaging of CBF and cerebral metabolic rate of glucose consumption (CMR_glc_) in the late 1970s and 1980s, models were the backbone of quantitative imaging by functional imaging technology. Without quantification by modeling, images of CBF and CMR_glc_ would be meaningless (although non-quantitative peripheral measures still play a role in some brain activation studies).

Fortunately, it was possible to present the many aspects of quantitative brain imaging according to the principles presented above. The quantification of brain images has undergone an impressive development, from the onset of functional mapping of blood flow to the most complex mapping of second and third messenger responses to neurotransmission. Obtaining images through appropriate instrumentation, such as PET or MRI, is the important first step in quantification. The recording often results in a dynamic series of images, although there are also simplified procedures for further clinical practical use. The final step is the assignment of quantitative values to neurobiological processes relevant to specific brain regions.

The application of graphical methods to the quantification of brain uptake of tracers in studies by means of PET or SPECT had many advantages and can be complementary to, or even replace, non-graphical compartmental modeling. The first approaches were borne out of attempts to quantify tracer influx without the need for computational methods that were not widely available in the early 1980s. The first plots were used widely in imaging, following the first applications to tissue sampling in rodent biodistribution studies (Gjedde, 1981, 1982; Patlak et al., 1983).

### Subdivisions of neurokinetics

In the Modeling sections, we considered graphical analysis of brain uptake of tracers in relation to arterial concentrations, and we considered the graphical analyses of tracer uptake in the brain in relation to regions with no specific binding or metabolism, identified as the regions of reference. We then showed the results of the analysis in terms of graphs indicative of occupancy of the tracer molecules at receptors or after transport by specific transporters or metabolic conversion by enzymes. Finally, we exemplified the results of analyses completed when tracer quantities in arterial blood or plasma or a known region or area of reference were unknown because the users measured no such quantities or knew the quantities not to be available or to exist.

The onset of neurotransmitter and receptor imaging in the early 1980s necessitated more sophisticated models of receptor-ligand interaction that distinguished specific from non-specific binding of radiolabeled ligands or drugs. The application of the models required several measurements of the actions of neurotransmitters in the synapse and extracellular space after activation of brain functions. Due to the many novel approaches to the mapping of functional correlations of neuroanatomy and neurotransmission that appeared in the last decades, we now find that it is necessary to further refine and present the challenges to the formulation of the mathematical models used by researchers.

In most cases, we assigned the tissue components by reference to known neuroanatomical subsections such as regions (ROIs) or volumes (VOIs) of interest or by groups of volume elements (known as voxels) indicated by independent statistical parametric mapping (SPM). The voxels refer to the effects of specific phenomena or stimuli at sites that we generally know to represent networks of cooperating neuronal ensembles and brain regions. Regardless of the approach, the task always depends on some form of a quantitative model. In the present publication, we focused on assigning physiological values to the models, especially the amount of receptor or transporter proteins per unit volume of brain tissue.

By definition, modeling simplifies the understanding of a physiological or pathological process itself. In the case of measures of brain function, two approaches are common. They cover the widely used compartmental model and the less known but often relevant non-compartmental models. While the latter models are particularly relevant to more specialized measurements, such as the assessment of heterogeneity of blood flow levels (e.g., Kuikka et al., 1991), we here confined the presentation to the more traditional and common compartment model.

### Endogenous neurotransmitters

Interventions or changes in brain conditions that affect the release and concentration of endogenous neurotransmitters are known to affect the binding of radioligands at tracer doses without affecting the state of the receptors represented by the estimates of the binding parameters *B*_max_ and V_*d*_*K*_*D*_ shown above. The steps involved in the formulations of the necessary equations showed the multiple factors that interact in the process of binding a tracer ligand, as expressed in the magnitude of the binding potential. The binding potential depends not only on the number of receptors but also on the arterial concentration, volumes of distribution, and affinity of any exogenous ligand, as well as the concentration and affinity of the endogenous ligand or any endogenous inhibitor of the binding, as expressed in the equation,


(69) $$B\,P_{N\,D}=\frac{B_{\text{max}}}{V_{e}\,C_{a}+V_{d}\,K_{D}\,\left(1+\frac{C_{I}}{K_{I}}\right)}$$

where *C*_*a*_ is the arterial concentration of the tracer ligand, *V*_*e*_ is the exchange volume, *K*_*D*_ is the half-saturation constant of the tracer ligand, *C*_*I*_ is the concentration of the competing endogenous ligand, and *K*_*I*_ is the half-saturation concentration of the competing endogenous ligand. We now know intervention into a brain condition to affect the binding potential of a tracer ligand without overtly affecting the maximum binding capacity, the volume of distribution, or the affinity, of the receptors for the exogenous ligand. In these cases, we may infer a change of the concentration or inhibitory constant of an endogenous competitor when studying the effect of dopamine on the binding of the relevant radioactive tracer,


(70) $$\frac{C_{I}}{K_{I}}=\frac{B_{\text{max}}}{\left[V_{d}\,K_{D}\right]\,\left[B\,P_{N\,D}\right]}-\left(\frac{\left[V_{e}\,C_{a}\right]}{\left[V_{d}\,K_{D}\right]}+1\right)$$

where *C*_*I*_ is the concentration of an endogenous inhibitor, the magnitude of which we can calculate by subtraction. The change of concentration then takes the form,


(71) $$\frac{\Delta \,C_{I}}{K_{I}}=\frac{B_{\text{max}}}{\left[V_{d}\,K_{D}\right]}\,\left(\frac{B\,P_{N\,D}^{b}-B\,P_{N\,D}^{c}}{B\,P_{N\,D}^{b}\,B\,P_{N\,D}^{c}}\right)=\frac{B\,P_{N\,D}^{a}}{B\,P_{N\,D}^{b}}\,\left(\frac{B\,P_{N\,D}^{b}}{B\,P_{N\,D}^{c}}-1\right)$$

where the letters a, b, and c refer to binding potentials in the absence of any occupant (a), at the experimental baseline (b), and in the experimental state (c), the last two before and after the procedure or change in the state of the brain. We often refer to the term in parenthesis $\left(\left[B\,P_{N\,D}^{b}/B\,P_{N\,D}^{c}\right]-1\right)$ as the fractional transmitter “release,” although its size depends on the transmitter occupation of the receptors at baseline, and although release is only one of many possible interpretations.

Dopaminergic neurotransmission consistent with dopamine release has been reported in a number of situations, the most common being the effect of amphetamine administration and non-drug intervention in experiments of exposure of individuals to neuropsychological stimuli (Koepp et al., 1998; Gjedde et al., 2010). The evidence includes former cocaine addicts who re-expose themselves to cocaine-related cues (Wong et al., 2006), as illustrated in Figure 2. Some reported changes differ in healthy volunteers and patients with neuropsychiatric disorders (Laruelle and Abi-Dargham, 1999; Wong et al., 2008). Evidence of dopamine release also addressed other transmission systems, including noradrenergic (McConathy et al., 2001) and opioid (Maarrawi et al., 2007; Scott et al., 2007) transmission systems. The method also relates to transmitter depletion, such as inhibition of dopamine synthesis by α-methyl-β-tyrosine, which caused relevant binding potentials to increase (Laruelle et al., 1997).

### Challenges to quantification

The greatest current challenge to quantification of receptor binding parameters is the interpretation of results of regression analysis, from model-based convolution results to observed data. Progress in this area improved and enormously simplified the calculation. The stochastic nature of brain imaging fundamentally affects the quality of observed data. This was true when the signal varied throughout the three-dimensional volumes under the influence of the physical process of generating two photons and the partial volume effects of the tomography itself, related to the inhomogeneity of tissue composition(s). These factors lead to significant variance of model parameters with most partial volume methods and thus introduce uncertainty into the estimation of parameters of the relevant equations (Wong and Gjedde, 1996).

For good reasons, this discussion does not address future quantification challenges, including corrections for finite instrument resolutions (e.g., partial volume effects), absence of sufficiently specific radiopharmaceuticals (e.g., tracers that attract multiple receptor sites), and variable degrees of radiotracer metabolism. Despite these concerns, in this article, we have attempted to provide a general overview of mathematical modeling approaches to functional brain imaging by means of PET or SPECT as we wait for more novel and increasingly challenging but exciting imaging modalities available to the practice of functional brain research.

### Limitations of reference region methods

The evidence presented in the section of Modeling by regional reference region shows that there are explicit limitations of common reference methods such as SRTM (Lammertsma et al., 1996), Logan (Logan et al., 1996), and MRTM2 (Ichise et al., 2003) in terms of the accuracy of the calculation of bound vs. free concentrations when d*B*/dt = 0. The current reference tissue methods generally do not allow the precise determination of the free concentration of radiotracer at the time when the change of the concentration of bound tracer (*B*) importantly is zero. For all short-lasting bolus injections of the radiotracer, there typically is only one instance of time when the free and bound quantities can be used to determine the correct binding potential. Methods of estimating *B*_max_ and *K*_D_ with reversibly binding radioligands with definite reference regions may suffer from this uncertainty. The method of TREMBLE that solves the problem has been presented in considerable detail, justified by the precise determination when d*B*/dt = 0.

## Summary

Here we attempted to provide a unified description of kinetic modeling with and without arterial input functions, as it evolved in the last 40 years, from the historical origins of deoxyglucose-based tracers of glucose metabolism to the development of estimates of binding potential, absolute receptor density, and apparent affinity of neuroreceptor systems measured *in vivo* by PET. The remaining and future challenges include appropriate interpretations of the current methods of estimating reasonable model parameters from variably proposed models.

## Data availability statement

The original contributions presented in this study are referenced in the article. Further inquiries can be directed to the corresponding author/s.

## Author contributions

Both authors listed have made a substantial, direct, and intellectual contribution to the work, and approved it for publication.
